# Symbiotic combination of *Akkermansia muciniphila* and inosine alleviates alcohol-induced liver injury by modulating gut dysbiosis and immune responses

**DOI:** 10.3389/fmicb.2024.1355225

**Published:** 2024-03-20

**Authors:** Li Wei, Yizhi Pan, Yu Guo, Yin Zhu, Haoran Jin, Yingying Gu, Chuanshuang Li, Yaqin Wang, Jingjing Lin, Yongping Chen, Chunhai Ke, Lanman Xu

**Affiliations:** ^1^Department of Infectious Diseases and Liver Diseases, Ningbo Medical Center Lihuili Hospital, Affiliated Lihuili Hospital of Ningbo University, Ningbo, China; ^2^Hepatology Diagnosis and Treatment Center, The First Affiliated Hospital of Wenzhou Medical University & Zhejiang Provincial Key Laboratory for Accurate Diagnosis and Treatment of Chronic Liver Diseases, Wenzhou, China; ^3^Department of Infectious Diseases, Taizhou Enze Medical Center (Group), Enze Hospital, Taizhou, China

**Keywords:** alcoholic liver disease, *Akkermansia muciniphila*, inosine, gut microbiota, adenosine 2A receptor, Regulatory T Cells

## Abstract

**Background:**

Alcoholic liver disease (ALD) is exacerbated by disruptions in intestinal microecology and immune imbalances within the gut–liver axis. The present study assesses the therapeutic potential of combining *Akkermansia muciniphila* (*A. muciniphila*) with inosine in alleviating alcohol-induced liver injury.

**Methods:**

Male C57BL/6 mice, subjected to a Lieber-DeCarli diet with 5% alcohol for 4 weeks, served as the alcoholic liver injury model. Various analyzes, including quantitative reverse transcription polymerase chain reaction (qRT-PCR), ELISA, immunochemistry, 16S rRNA gene sequencing, and flow cytometry, were employed to evaluate liver injury parameters, intestinal barrier function, microbiota composition, and immune responses.

**Results:**

Compared to the model group, the *A. muciniphila* and inosine groups exhibited significantly decreased alanine aminotransferase, aspartate aminotransferase, and lipopolysaccharide (LPS) levels, reduced hepatic fat deposition and neutrophil infiltration, alleviated oxidative stress and inflammation, and increased expression of intestinal tight junction proteins (Claudin-1, Occludin, and ZO-1). These effects were further pronounced in the *A. muciniphila* and inosine combination group compared to individual treatments. While alcohol feeding induced intestinal dysbiosis and gut barrier disruption, the combined treatment reduced the abundance of harmful bacteria (*Oscillibacter, Escherichia/Shigella, and Alistipes*) induced by alcohol consumption, promoting the growth of butyrate-producing bacteria (Akkermansia, Lactobacillus, and Clostridium IV). Flow cytometry revealed that alcohol consumption reduced T regulatory (Treg) populations while increasing those of T-helper (Th) 1 and Th17, which were restored by *A. muciniphila* combined with inosine treatment. Moreover, *A. muciniphila* and inosine combination increased the expression levels of intestinal CD39, CD73, and adenosine A2A receptor (A2AR) along with enhanced proportions of CD4^+^CD39^+^Treg and CD4^+^CD73^+^Treg cells in the liver and spleen. The A2AR antagonist KW6002, blocked the beneficial effects of the *A. muciniphila* and inosine combination on liver injury in ALD mice.

**Conclusion:**

This study reveals that the combination of *A. muciniphila* and inosine holds promise for ameliorating ALD by enhancing the gut ecosystem, improving intestinal barrier function, upregulating A2AR, CD73, and CD39 expression, modulating Treg cells functionality, and regulating the imbalance of Treg/Th17/Th1 cells, and these beneficial effects are partly A2AR-dependent.

## 1 Introduction

Alcoholic liver disease (ALD) encompasses alcoholic fatty liver disease, alcoholic hepatitis, alcoholic liver fibrosis, cirrhosis, and hepatocellular carcinoma. Globally, in 2020, approximately 1.34 billion men and 312 million women engaged in harmful alcohol consumption, leading to approximately 1.78 million deaths attributed to alcohol consumption (Cabezas, 2022; Huang et al., 2023). Reports indicate that 50% of cirrhosis-related mortality is linked to alcohol consumption, which poses a significant threat to human health (Xiao et al., 2019; Avila et al., 2020).

The primary morphological characteristics of ALD include steatosis, hepatocyte ballooning degeneration, necrosis, and lobular inflammation primarily characterized by neutrophil polymorphism. With disease progression, inflammatory and fibrotic alterations may extend to the hepatic veins, eventually advancing to the stage of micronodular cirrhosis (Addolorato et al., 2020a). Dysbiosis of the intestinal microbiota is a pathogenic mechanism of ALD that results inintestinal barrier dysfunction, altered microbiota, and immune cell changes (Ge et al., 2019, 2022). An increase in intestinal permeability, resulting from a breached intestinal barrier, gut-derived bacteria and products, such as LPS, the main component of endotoxin, can bind to Toll-like receptor 4 (TLR4), increasing the recruitment of Myd88 and ultimately accelerating the nuclear translocation of NF-κB. This activation induces the expression and release of multiple inflammatory factors, which stimulate the innate immune system and damage the liver (Albillos et al., 2020; Siddiqui and Cresci, 2020). Metabolic components produced by the intestinal microbiota, such as short-chain fatty acids, amino acids, and their derivatives, act on intestinal epithelial cells and directly or indirectly affect various immune cells of the mucosal immune system. ALD leads to marked immune cell imbalances, such as the Treg/Th17 cells ratio, which is closely associated with gut microbial dysbiosis in intestinal microecology (Gao and Bataller, 2011; Szabo, 2015). Thus, modulating intestinal microecology and barrier function is crucial for maintaining immune homeostasis in the gut–liver axis.

*Akkermansia muciniphila,* a strictly anaerobic Gram-negative bacterium, is the only known member of the human gut-associated phylum Verrucomicrobia, comprising approximately 0.5–5% of the human intestinal microbiota (Cani et al., 2022, 2023). Its abundance is negatively correlated with various diseases, including inflammatory bowel disease, neurodegenerative diseases, obesity, and autism (Rodrigues et al., 2022). Notably, *A. muciniphila* can upregulate the RORγt^+^ Treg cell-mediated immunosuppressive response by interacting with TLR4, which plays a key role in intestinal mucosal homeostasis in mice (Liu et al., 2022). The influence of *A. muciniphila* on host physiology depends, in part, on the small molecules produced through the joint metabolism of the host and microbial community. One such metabolite, inosine, serves as a physiological energy source and exhibits potent anti-inflammatory and immunomodulatory properties (Saveljeva et al., 2022). A previous study revealed that pretreatment with inosine reduced acute liver damage and inflammation, in part by controlling the TLR4/NF-κB signaling pathway (Guo et al., 2021). However, the additional benefits and underlying mechanisms of the combination of *A. muciniphila* and inosine in ALD remain unclear.

One of the four adenosine receptors in the G-protein-coupled receptor superfamily, the adenosine 2A receptor (A2AR), exhibits strong anti-inflammatory properties that affect immune cell regulation (Haskó et al., 2008). The ectoenzymes CD39 and CD73, which are highly expressed in Tregs, hydrolyze the extracellular ATP (eATP) produced in injured tissues to adenosine (ADO), thereby reducing inflammation. Notably, the proliferation and maintenance of Tregs are related to the expression of CD39 and CD73. A2AR agonists significantly increase Treg numbers and enhance their immunosuppressive function (Haskó et al., 2008; Xing et al., 2023). Moreover, A2AR activation alleviates intestinal inflammation in inflammatory bowel diseases, potentially by promoting interleukin (IL)-10 and transforming growth factor-beta (TGF-β) secretion (Odashima et al., 2005). In A2AR-knockout models, IL-10 and TGF-β levels are significantly decreased, accompanied by an increase in interferon-gamma (IFN-γ) levels (Nowak et al., 2010). These cytokines play important roles in the regulation of Treg, Th17, and Th1 cell differentiation. These data suggest that the adenosine A2AR pathway may be involved in the differentiation and function of immune cells to maintain immune homeostasis in the gut–liver axis; however, this hypothesis requires verification.

In the present study, we investigated the potential protective effects of *A. muciniphila* combined with inosine in ALD mice. We analyzed the intestinal microbiota components, mucosal barrier, and the balance of Treg/Th17/Th1 in the liver and spleen. To further explore the potential mechanisms, we measured the levels of CD39, CD73, and A2AR in the intestine as well as the proportions of CD39^+^ Tregs and CD73^+^ Tregs in the liver and spleen. Our findings demonstrate that *A. muciniphila* combined with inosine may be a promising therapy for patients with ALD.

## 2 Materials and methods

### 2.1 *Akkermansia muciniphila* culture

The *A. muciniphila* strain acquired from the American Type Culture Collection (ATCC Number: BAA-835) was cultured in an anaerobic environment at 37°C using brain-heart infusion (BHI) (CM1135B, OXOID) liquid medium supplemented with 0.5% (wt/vol) mucin (M2378, Sigma). The cultures were incubated for 48 h, centrifuged (3,500 rpm, 10 min, 4°C), washed twice in sterile saline, and resuspended at 5 × 10^9^ CFU/mL (measured by absorbance at 630 nm) for intragastric injection (Grander et al., 2018).

### 2.2 Animals and treatment

Six to eight-week-old male C57BL/6 mice were procured from Beijing Vital River Laboratory Animal Technology Co., Ltd. and housed in an SPF environment. The ALD model was developed as previously described (Zhu et al., 2022). As shown in Supplementary Figure S2, mice were first fed a liquid control diet called Lieber-DeCali (Trophic Animal Feed High-tech Co., Jiangsu, China) for 1 week, after which they were fed an adaptive alcohol diet for 1 week (the ratio of Lieber-DeCarli control diet to ethanol diet was adjusted from 2:1 1:1 to 1:2 on days 2, 4 and 6, respectively). Following the acclimation phase, the mice were given a four-week supply of the full Lieber-DeCarli ethanol diet with 5% alcohol.

To investigate the effect of *A. muciniphila* combined with inosine on ALD, mice were randomly divided into five groups: (1) normal group (N, *n* = 10), maintained on a Lieber-DeCarli control diet until euthanasia; (2) model group (M, *n* = 10); (3) *A. muciniphila* treatment group (AKK, *n* = 10); (4) inosine (I4125, Sigma-Aldrich, St. Louis, MO, United States) treatment group (I, *n* = 10); (5) and the *A. muciniphila* and inosine combination group (AKK + I, *n* = 10). To investigate whether therapeutic effects of the combined intervention are mediated via A2AR, the ALD model animals were randomized into three groups: (1) the A2AR antagonist group (KW6002, *n* = 5), (2) the *A. muciniphila* and inosine combination group (AKK + I, *n* = 5), (3) and combination therapy +A2AR antagonist group (AKK + I + KW6002, *n* = 5).

For treatment with *A. muciniphila,* mice were gavaged with 1 × 10^9^ CFU *A. muciniphila* suspended in 0.2 mL of sterile saline every other day for 4 weeks on a complete ethanol diet. For treatment with inosine, mice were gavaged inosine (300 mg/kg) every other day for 4 weeks on a complete ethanol diet. For treatment with KW6002, mice were intraperitoneally injected with 5 mg/kg/day of KW6002 during the last 2 weeks of the complete ethanol diet. Sterile saline was used as a negative control for intragastric or intraperitoneal injections.

At the end of the experiment, all mice were euthanized, and blood, liver, spleen, fecal, and intestinal tissues were collected. The study protocols were approved by the Ethics Committee of the Health Science Center of Ningbo University (approval number 11417).

### 2.3 Liver and intestine histopathology

Samples of the liver and small intestine were obtained, fixed for 24 h in 10% formalin, and embedded in paraffin. Hematoxylin and eosin were used to stain liver and small intestine sections for pathological evaluation. Standard protocols were followed for the immunohistochemical studies (Zhu et al., 2022). Sections immersed in paraffin were deparaffinized and rehydrated. Liver sections were incubated with anti-MPO (PA5-16672, 1:200 dilution; Thermo Fisher Scientific, Waltham, MA, United States) and anti-F4/80 (SP115, 1:100 dilution; Abcam, Cambridge, United Kingdom) antibodies to assess macrophage and neutrophil infiltration, respectively. Small intestinal sections were incubated with anti-CD39 (ab223842, 1:1000 dilution; Abcam), anti-CD73 (ab288154, 1:500 dilution; Abcam), and anti-A2AR (ab3461, 1:200 dilution; Abcam) antibodies to evaluate the levels of intestinal CD39, CD73, and A2AR proteins. PE-coupled secondary anti-rabbit IgG was used for detection. Liver and small intestine sections were rapidly frozen, cut into 4-μm sections, and stained with Oil Red O. Images were observed with a clear field microscope (Nikon, Tokyo, Japan) and analyzed using Image-Pro Plus 6.0 software (Media Cybernetics, Rockville, MD, United States). Each specimen was randomly selected with three independent visual fields at 20× magnification. Liver hepatic steatosis was evaluated using the Brunt scale by a pathologist who was blinded to the study.

### 2.4 Serum biochemical analysis and ELISA assay

An automatic biochemical analyzer (AU5800; Beckman Coulter, Brea, CA, USA) was used to measure serum alanine aminotransferase (ALT) and aspartate aminotransferase (AST) levels. ELISA kits (MultiSciences, China) were used to measure the levels of hepatic IL-17A, IFN-γ, and IL-10 proteins, in accordance with the manufacturer’s instructions. ELISA kits (Nanjing Jiengcheng Bioengineering Institute, China) were used to measure the levels of hepatic glutathione (GSH), oxidized glutathione (GSSG), and superoxide dismutase (SOD), in accordance with the manufacturer’s instructions. ELISA kits (MyBioSource) were used to measure the serum levels of LPS, following the manufacturer’s instructions.

### 2.5 Real-time reverse transcriptase polymerase chain reaction (RT-PCR) assay

The RNA Extraction Kit (74,104; QIAGEN, Hilden, Germany) was used to extract total RNA, and the PrimeScript RT Reagent Kit (RR047A, Takara Bio, Shiga, Japan) was used to reverse-transcribe the extracted RNA into cDNA. TB Green Premix Ex Taq (RR420A; Takara Bio) was used to amplify cDNA. A Thermo Fisher QuantStudio5 real-time PCR system was used to quantify the amounts of RNA. The 2^-ΔΔCt^ method was used to assess the final data, and glyceraldehyde 3-phosphate dehydrogenase (*GAPDH*) was used as the reference gene for mRNA expression estimates. The primer sequences are listed in Supplementary Table S1.

### 2.6 Western blot analysis

Small intestinal tissues were homogenized in RIPA buffer (Solarbio, China) with a mixture of protease inhibitors. The Pierce BCA Protein Assay Kit (TransGen Biotech, China) was used to quantify protein concentration. Protein samples were subjected to electrophoresis using on 10% sodium dodecyl sulfate-polyacrylamide gels and transferred to PVDF membranes (MilliporeSigma, Burlington, MA, United States). Subsequently, the membranes were blocked with skimmed milk powder (Solarbio) and incubated overnight at 4°C with the following primary antibodies: anti-ZO-1 (ab96587, 1:1000 dilution; Abcam), anti-Occludin (A2601, 1:2000 dilution; ABclonal, Woburn, MA, United States), anti-Claudin-1 (ab307692, 1:1000 dilution; Abcam:1000), and anti-GAPDH (ab181602, 1:1000 dilution; Abcam). After five washes with TBST for 5 min each, the membranes were incubated with the respective secondary antibodies (goat anti-rabbit; SA00001-2, Proteintech, Rosemount, IL, United States) for 1 h at room temperature. Protein bands were visualized using a high-sensitivity ECL chemiluminescence kit (NCM Biotech, China) and gel imager (Bio-Rad Laboratories, Hercules, CA, USA), and band intensity was quantified using ImageJ software version 2.3.0 (National Institute of Health, Bethesda, MD, USA).

### 2.7 16S rRNA gene sequencing analysis and bioinformatics analysis

Feces were immediately collected and stored at −80°C. Bacterial DNA was extracted using a FastDNA SPIN Kit for Soil (MP Biomedicals, Santa Ana, CA, USA) according to the manufacturer’s instructions. The V3-V4 hypervariable regions of the 16S rRNA gene, along with spike-ins, were amplified using primers 341F (5′-CCTACGGGNGGCWGCAG-3′) and 805R (5′-GACTACHVGGGTATCTAATCC-3′) and subsequently sequenced on an Illumina NovaSeq 6,000 sequencer (Illumina, San Diego, CA, United States) (BioProject ID PRJNA1071471). Usearch software was used to perform similarity level clustering analysis of reads, and reads with similarity greater than or equal to 97% were clustered into one operational taxonomic units (OTUs); RDP database was used as a reference database to taxonomically annotate the obtained OTUs. Principal Coordinate Analysis (PCoA) based on Unifrac distance was used to identify the microbial structure. Linear discriminant analysis (LDA) coupled with effect size measurement (LefSe) analysis was used to identify the microbial taxa with different degrees of enrichment in different treatment groups. The Kruskal-Wallis test (*p* < 0.05) was used to identify microbial taxa that differed among multiple groups.

### 2.8 Short-chain fatty acids (SCFAs) identification and quantification

Gas chromatography–mass spectrometry (GC–MS) was used to quantify typical SCFA for targeted metabolomics. Fecal samples were homogenized, centrifuged, filtered, and suspended in ultrapure water. Centrifugation was used to separate the ethyl acetate layer from the filtrate, and an equal volume of ethyl acetate was combined and incubated for 30 min at 4°C. SCFA standards at various concentrations were subjected to the same procedure. The SCFAs in the samples were characterized by comparing their retention times with those of the standards. A calibration curve based on the peak areas of the standards at different concentrations facilitated the quantification of SCFA levels.

### 2.9 Isolation of intrahepatic lymphocytes (IHL) and flow cytometry analysis

IHLs were isolated using a previously established method (Xu et al., 2021). Briefly, following anesthesia, the mouse liver was perfused with PBS. Liver tissues were collected and digested in RPMI-1640 medium containing collagenase IV (0.05%; C4-BIOC; Sigma-Aldrich) at 37°C for 30 min. To obtain single-cell suspensions, the digested cell suspensions were filtered through 70-μm nylon cell strainers. Centrifugation (400 × *g*) at room temperature for 30 min across a 30/70% discontinuous Percoll gradient was used to isolate IHLs (17,089,102; Cytiva). At interphase, cells were harvested, carefully washed, and resuspended in full RPMI-1640 medium supplemented with 10% FBS.

Regulatory T Cells were assayed using a Mouse Regulatory T Cell Staining Kit (88–8,111-40; Thermo Fisher Scientific), according to the manufacturer’s instructions. For Th1 and Th17 cell analysis, lymphocytes were first incubated with Leukocyte Activation Cocktail (550,583; BD Pharmingen, Franklin Lakes, NH, United States) for 5 h, followed by staining with Fixable Viability Stain 780 (565,388; BD Pharmingen), CD3e FITC (553,061; BD Pharmingen), CD4 APC (553,051; BD Pharmingen), and CD8a PerCP-Cy5.5 (551,162; BD Pharmingen) at 4°C for 30 min in the dark. A Fixation/Permeabilization Kit (554,714; BD Pharmingen) was used for fixation and permeabilization, followed by staining with IL-17A PE (55,952; BD Pharmingen) and IFN-γ PE (55,442; BD Pharmingen) at 4°C for 1 h in the dark. Samples were processed on a FACS Symphony A1 and analyzed using FlowJo X software version 10 (Tree Star, Ashland, OR, United States).

### 2.10 Statistical analysis

Statistics and graphing were performed using GraphPad Prism 8 (San Diego, CA, USA). One-way analysis of variance followed by Tukey’s multiple comparison test was used for continuous variables with normal distribution for comparison among multiple groups, and Kruskal-Wallis test was used for continuous variables with skewed distribution for comparison among multiple groups. Multiple comparisons were corrected by the Benjamini-Hochberg method, and *p* < 0.05 or false discovery rate (FDR) <0.05 were considered statistically significant differences. Data are presented as the mean ± SEM.

## 3 Results

### 3.1 Combination of *Akkermansia muciniphila* and inosine alleviated liver injury in ALD mice

**Figure 1:**
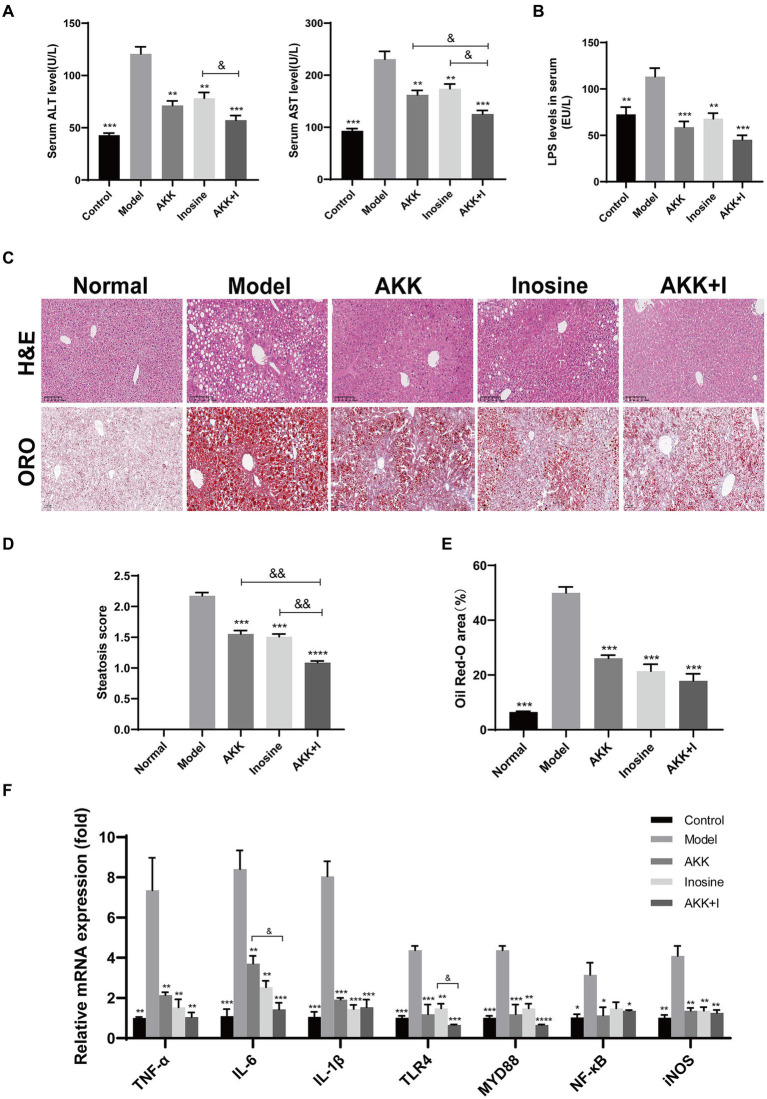
The combination of *Akkermansia muciniphila* and inosine alleviated liver injury in ALD mice. C57BL/6 mice were fed a Lieber-DeCarli diet containing 5% alcohol for 4 weeks. *A. muciniphila* (1 × 10^9^ CFU/mouse), inosine (300 mg/kg), or a combination of *A. muciniphila* (1 × 10^9^ CFU/mouse) and inosine (300 mg/kg) were administered orally every other day. All mice were euthanized in the 6th week. **(A)** Serum ALT and AST levels in different experimental groups. **(B)** The levels of LPS in the serum. **(C)** Representative images of hematoxylin and eosin and Oil Red O staining of liver sections (scale bar, 100 μm). **(D,E)** Statistical analysis of steatosis scores and Oil Red O staining. **(F)** Fold changes in the mRNA levels of proinflammatory cytokines determined via qRT-PCR. Data are shown as the mean ± SEM; *p < 0.05, **p < 0.01, ***p < 0.001 compared with the model group; &*p* < 0.05, &&*p* < 0.01 compared with the AKK + I group. *N* = 10 for each group.

In this study, we established an alcoholic liver injury model using a Lieber-DeCarli ethanol diet (5% ethanol). The model group exhibited elevated serum ALT, AST and LPS levels (Figures 1A,B), hepatocyte ballooning, and fat deposition (Figures 1C–E), along with significant macrophage and neutrophil infiltration (Supplementary Figure S3), compared to the normal group. This result suggests that alcohol exposure induced significant liver injury and inflammation. Application of *A. muciniphila* or inosine alone attenuated ALT, AST and LPS levels (Figures 1A,B), decreased ethanol-induced fat accumulation (Figures 1C–E), and reduced macrophage and neutrophil infiltration in the liver (Supplementary Figure S3). Notably, the combination of *A. muciniphila* and inosine further alleviated hepatic fat accumulation and attenuated ALT and AST levels, compared to treatment with *A. muciniphila* or inosine alone. These findings highlight the synergistic effect of *A. muciniphila* and inosine in ameliorating liver damage induced by chronic ethanol consumption.

### 3.2 Combination of *Akkermansia muciniphila* and inosine alleviates oxidative stress and inflammation in ALD mice

Given that oxidative stress is implicated in the pathogenesis of ALD, we evaluated the levels of glutathione, GSSG, and SOD in the five groups to assess susceptibility to oxidative stress. The levels of GSH, GSH/GSSG, and SOD were significantly reduced in the model group. Treatment with *A. muciniphila* significantly restored GSH levels compared to those in the model group, and the GSH/GSSG ratio was restored in both the *A. muciniphila* and inosine individual treatment groups. Notably, these effects were further pronounced in the combination treatment group (Supplementary Figures S4A,C,D). However, there were no significant differences in GSSG levels among the five groups (Supplementary Figure S4B).

To evaluate liver inflammation, we detected changes in the expression of representative inflammatory markers at the transcriptional level. The mRNA expression levels of *TNF-α*, *IL-6*, *IL-1β*, *TLR4*, *Myd88*, *NF-κB*, and *iNOS* were significantly higher in alcohol-fed mice than in normal mice, indicating that alcohol consumption induced liver inflammation (Figure 1F). Treatment with either *A. muciniphila* group or inosine decreased the transcription levels of these above indicators. Treatment with the combination further reduced the transcriptional levels of *IL-6* and *TLR4* and improved those of *IL-10* and *AMPK* (Figure 1F and Supplementary Figure S5). However, no significant differences was observed for the other indicators (*IL-2*, *COX-2*, *IκB-α*, and *Nfr2*) (Supplementary Figure S5). These results suggest that the combination of *A. muciniphila* with inosine ameliorates alcohol-induced oxidative stress and the hepatic inflammatory response.

### 3.3 Combination of *Akkermansia muciniphila* and inosine restored gut barrier function in ALD mice

**Figure 2:**
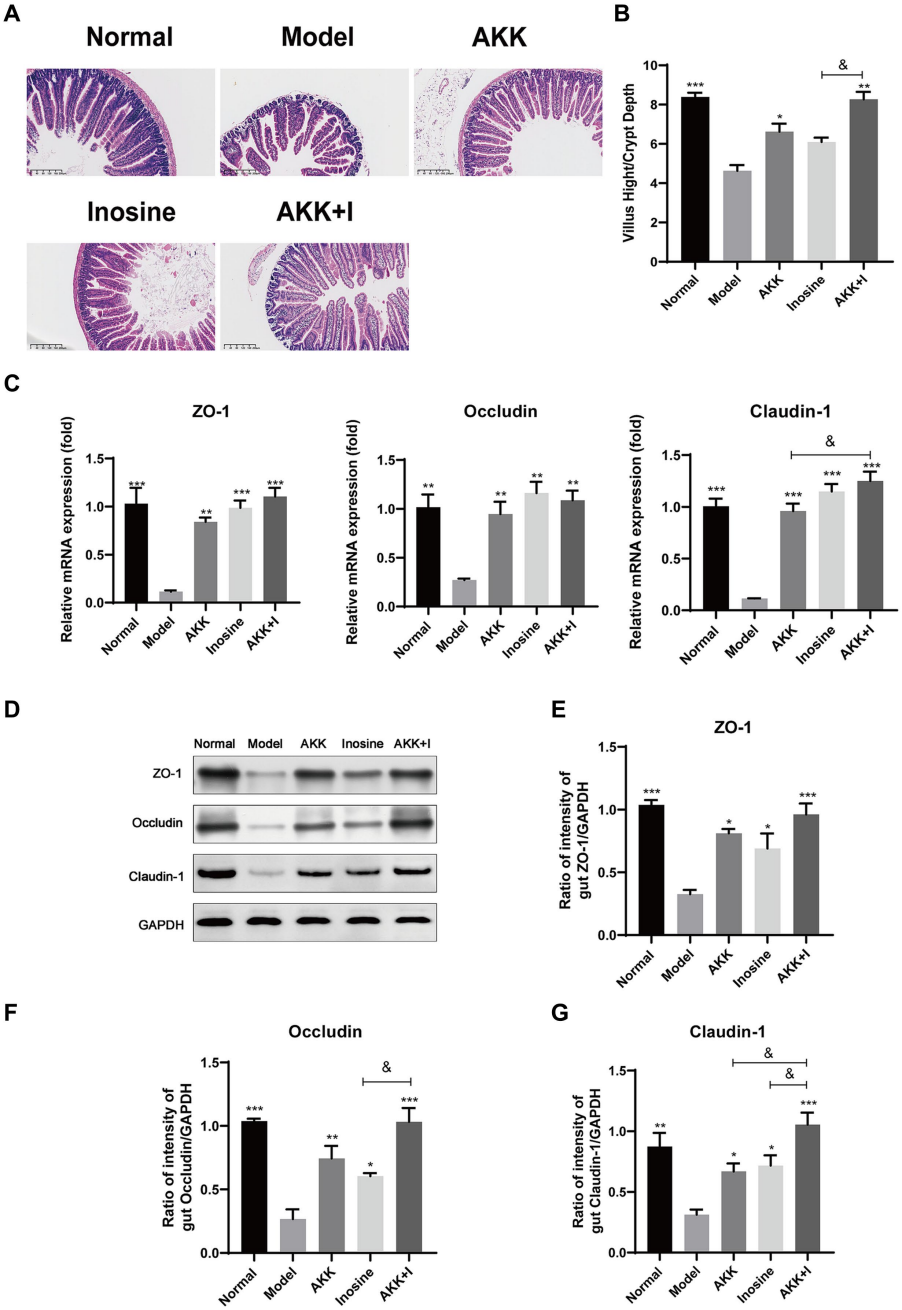
Improvement of intestinal barrier dysfunction with *Akkermansia muciniphila* and inosine treatment. ALD mice were treated as described in Figure 1. **(A)** Representative images of hematoxylin and eosin-stained histological sections of the small intestine (scale bar: 200 μm). **(B)** Measurements of the villi and crypts from three sections per group. **(C)** Fold changes in the mRNA levels of tight junction proteins determined via qRT-PCR. **(D–G)** representative Western blot images and histograms of the band densities of ZO-1, Occludin, and Claudin-1 in the small intestine of each experimental group. Data are shown as the mean ± SEM; *p < 0.05, **p < 0.01, ***p < 0.001 compared to the model group; &*p* < 0.05, &&*p* < 0.01 compared with the AKK + I group. *N* = 10 each group for hematoxylin and eosin-stained; *N* = 6 each group for RT-qPCR; *N* = 3 each group for Western blot.

Hematoxylin and eosin staining of mouse small intestines revealed irregular and shorter villi in the ALD model group compared to those in the normal group. Treatment with either *A. muciniphila* or inosine improved the villus lesions, and the *A. muciniphila* and inosine combination further increased the villus height-to-crypt depth ratio, indicating an improvement in villus–crypt connections during alcohol-induced barrier disruption (Figures 2A,B). Furthermore, we measured the expression of tight junction (TJ) proteins, including Claudin-1, Occludin, and ZO-1, in the small intestine. Compared with the normal group, alcohol feeding significantly reduced the mRNA expression of the genes encoding TJ proteins, whereas treatment with either *A. muciniphila* or inosine alone upregulated the expression of the genes. Notably, the combination treatment significantly restored Claudin-1 expression compared to that with *A. muciniphila* treatment alone (Figure 2C). The results of western blotting were consistent with the gene expression results; combination treatment with *A. muciniphila* and inosine further increased the expression levels of these TJ proteins compared to those in the individual treatment groups (Figures 2D–G). Overall, our findings suggest that the combined treatment with *A. muciniphila* and inosine improved intestinal barrier function in an ALD mouse model.

### 3.4 Combined treatment ameliorates alcohol-induced intestinal dysbiosis

**Figure 3:**
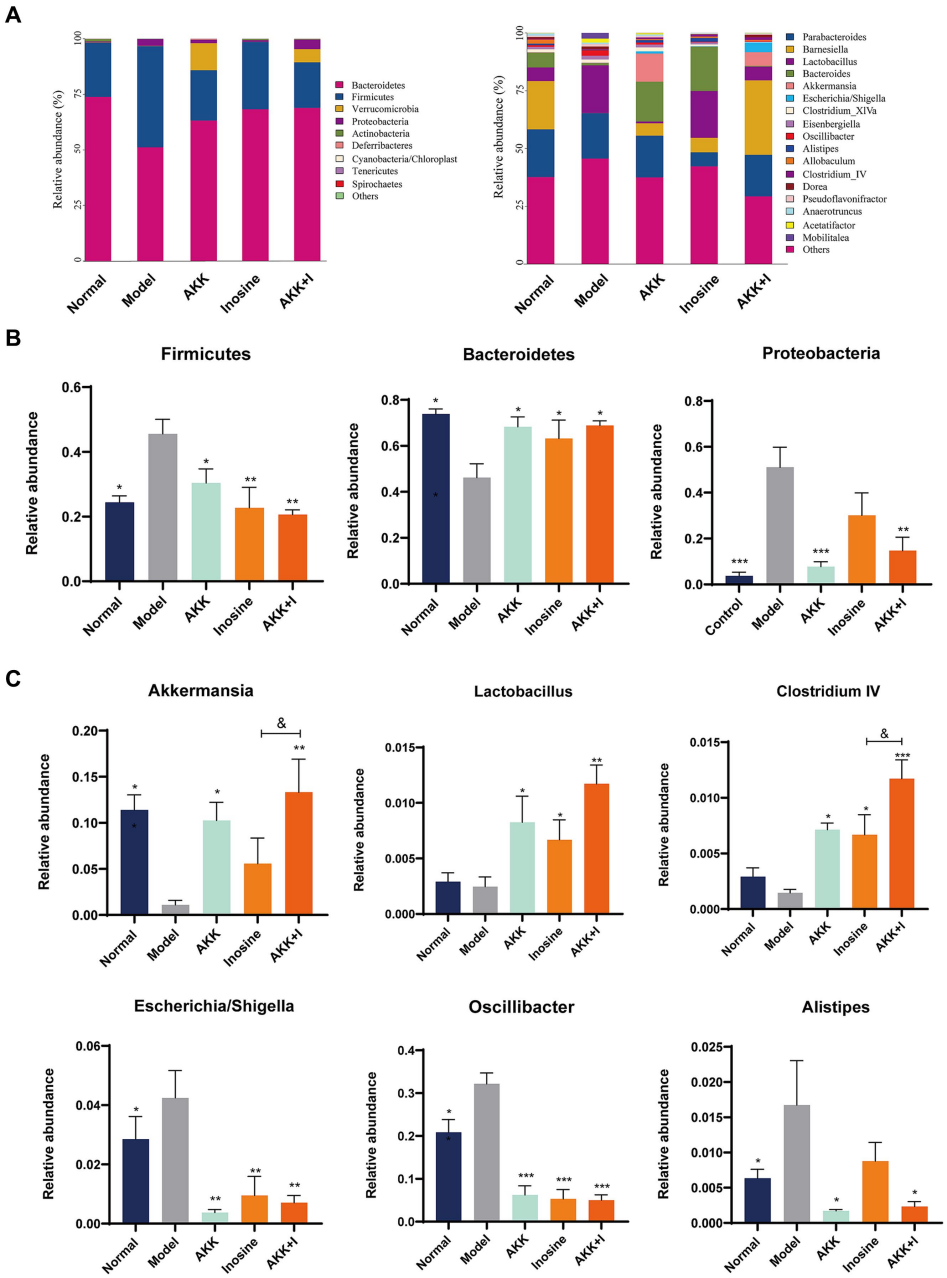
The combination of *Akkermansia muciniphila* and inosine regulates alcohol-induced intestinal dysbiosis. ALD mice were treated as described in Figure 1. **(A)** Relative abundance of predominant bacteria (>1% in each sample) at the phylum and genus levels. **(B)** Relative abundances of *Firmicutes, Bacteroidetes*, and *Proteobacteria* at the phylum level in feces. **(C)** The relative abundance of *Bacteroides*, *Lactobacillus*, *Clostridium* IV, *Oscillibacter*, *Escherichia*/*Shigella*, and *Alistipes* at the genus level in feces. Data are shown as the mean ± SEM; *p < 0.05, **p < 0.01, ***p < 0.001 compared to the model group; &*p* < 0.05, &&*p* < 0.01 compared with the AKK + I group. *N* = 6 for each group.

We performed 16S rRNA sequencing analysis to investigate the changes in the gut microbiota in the altered intestinal environment. The results revealed alterations in bacterial abundance at the phylum and genus levels due to alcohol exposure (Figure 3A). Alcohol feeding increased the abundance of *Firmicutes* and *Proteobacteria* but decreased the proportion of *Bacteroidetes* compared to that in the normal group (Figure 3B). However, the combination of *A. muciniphila* and inosine specifically increased *Bacteroidetes, Akkermansia, Lactobacillus*, and *Clostridium IV*, while decreasing the abundance of *Firmicutes*, *Proteobacteria*, *Oscillibacter*, *Escherichia*/*Shigella*, and *Alistipes* (Figures 3B,C). Wilcoxon analysis and LDA scores indicated that *Bacilli, Desulfovibrionaceae*, *Mobilitalea*, and *Peptococcaceae* were significantly associated with the model group. *Verrucomicrobiaceae* and *Akkermansia* spp. were significantly enriched in the Akkermansia group. *Rikenellaceae*, *Sutterellaceae*, and *Alistipes* were the dominant taxa in the inosine group. *Barnesiell*a, *Clostridium IV*, and *Candidatus_Saccharimonas* were enriched in the combination intervention group (Supplementary Figure S6). Overall, these results indicate that *A. muciniphila* combined with inosine effectively improved gut dysbiosis induced by ethanol feeding.

### 3.5 Combination of *Akkermansia muciniphila* and inosine increased SCFAs levels

**Figure 4:**
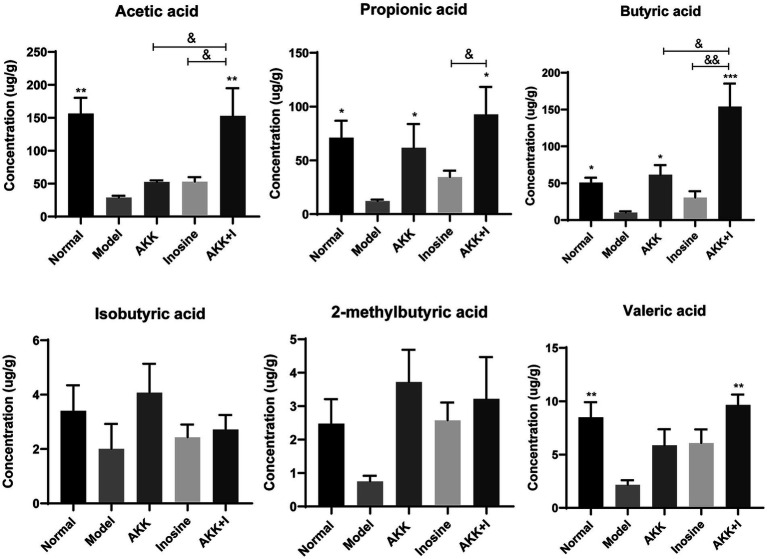
The combination of *Akkermansia muciniphila* and inosine improves SCFA production. ALD mice were treated as described in Figure 1. **(A)** Levels of SCFAs in fecal samples from different groups. Data are shown as the mean ± SEM; *p < 0.05, **p < 0.01, ***p < 0.001 compared to the model group; &*p* < 0.05, &&*p* < 0.01 compared with the AKK + I group. *N* = 6 for each group.

SCFAs are indispensable signaling molecules in the gut–liver axis. Therefore, we conducted targeted metabolomic analysis to determine the concentrations of specific SCFAs, including acetate, propionate, isobutyrate, butyrate, 2-methylbutyrate, and valerate, in mouse fecal samples. Alcohol consumption markedly reduced the concentrations of acetic, propionic, butyric, and valeric acids (Figure 4). Conversely, *A. muciniphila* administration increased the production of propionic and butyric acids. Inosine-treated mice exhibited elevated levels of acetic, propionic, and butyric acids; however, the difference was not significant compared to the model group (Figure 4). Importantly, in the combination group, the levels of acetate, butyrate, and valerate were significantly increased compared to those in the model group. However, no significant differences were observed in isobutyrate or 2-methylbutyrate levels among the groups (Figure 4). These results suggest supplementation with both *A. muciniphila* and inosine partially reversed the alcohol-induced reduction in SCFAs.

### 3.6 Combined treatment regulates the imbalance of immune cells

**Figure 5:**
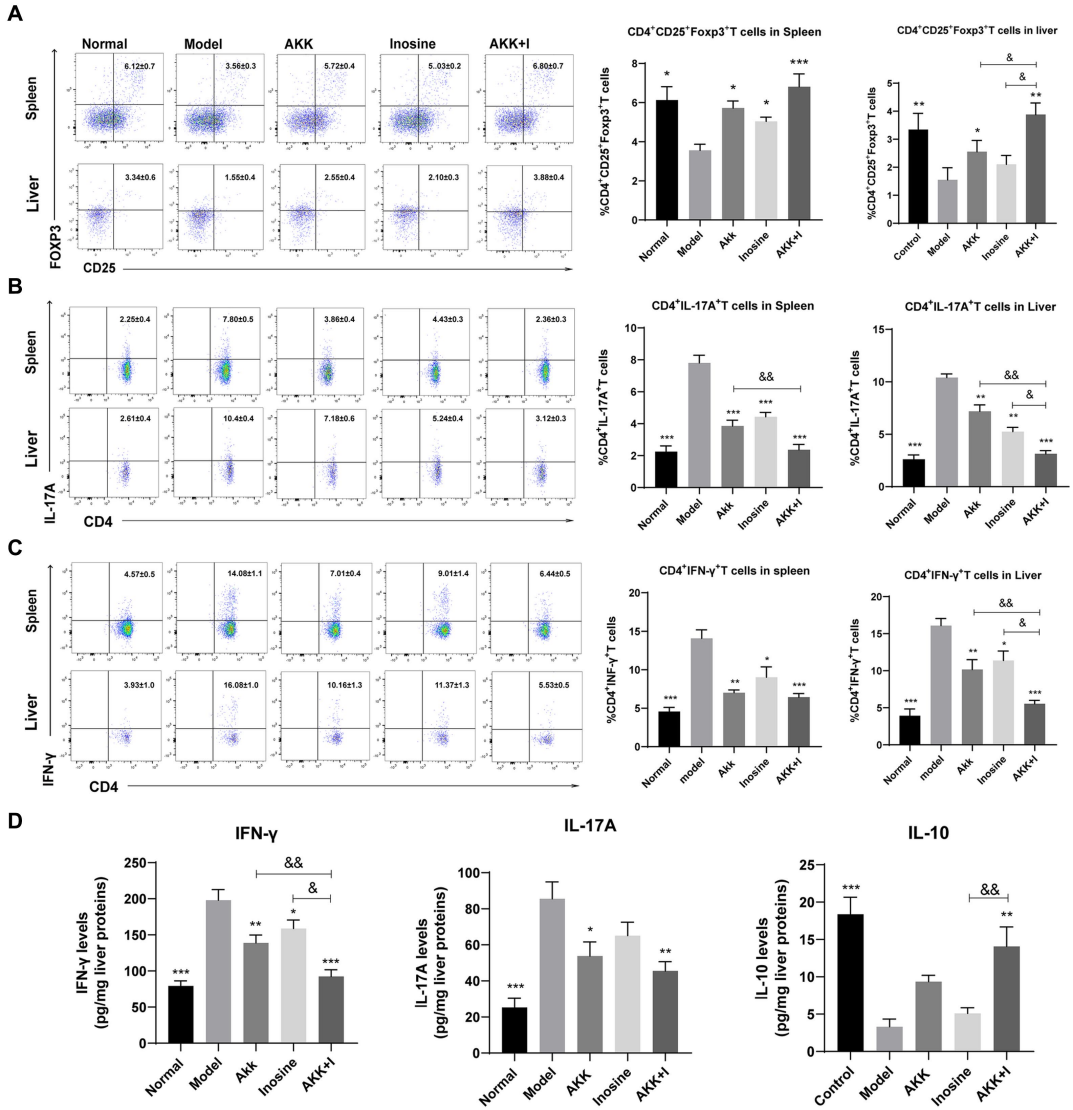
The combination of *Akkermansia muciniphila* and inosine reduces Th1 and Th17 cells but increases Treg cells in the spleen and liver. ALD mice were treated as described in Figure 1. **(A)** Representative flow cytometric plots and quantification of Tregs in the spleen and liver. **(B)** Representative flow cytometric plots and quantification of Th17 cells in the spleen and liver. **(C)** Representative flow cytometric plots and quantification of Th1 cells in the spleen and liver. **(D)** Protein levels of INF-γ, IL-17A, and IL-10 in the liver, as measured using ELISA. Data are shown as the mean ± SEM; *p < 0.05, **p < 0.01, ***p < 0.001 compared to the model group; &*p* < 0.05, &&*p* < 0.01 compared with the AKK + I group. *N* = 7–10 for each group.

We measured Treg, Th17, and Th1 cell numbers using flow cytometry and found that alcohol consumption decreased the proportion of Treg cells in the spleen and liver, while significantly increasing that of Th1 and Th17 cells (Figures 5A–C). Conversely, the administration of *A. muciniphila* or inosine alone increased Treg cell proportions, which were further increased by the combined *treatment with A. muciniphila* and inosine. The proportion of Th1 and Th17 cells in the *A. muciniphila* or inosine treatment groups was lower than that in the model group, and this reduction in the proportions was more pronounced with the combined treatment (Figures 5A–C). Consequently, intrahepatic IFN-γ and IL-17A levels were significantly elevated in the model group, whereas intrahepatic IL-10 levels were remarkably decreased compared to those in the normal group (Figure 5D). Notably, the combination of *A. muciniphila* and inosine reduced IFN-γ and IL-17A levels and restored IL-10 levels (Figure 5D). Therefore, the combined treatment inhibited liver inflammation by restoring the balance of dysregulated Th17/Th1/Treg immune responses.

### 3.7 Therapeutic effects of the combined *Akkermansia muciniphila* and inosine treatment might be mediated by CD39-CD73-A2AR pathway

**Figure 6:**
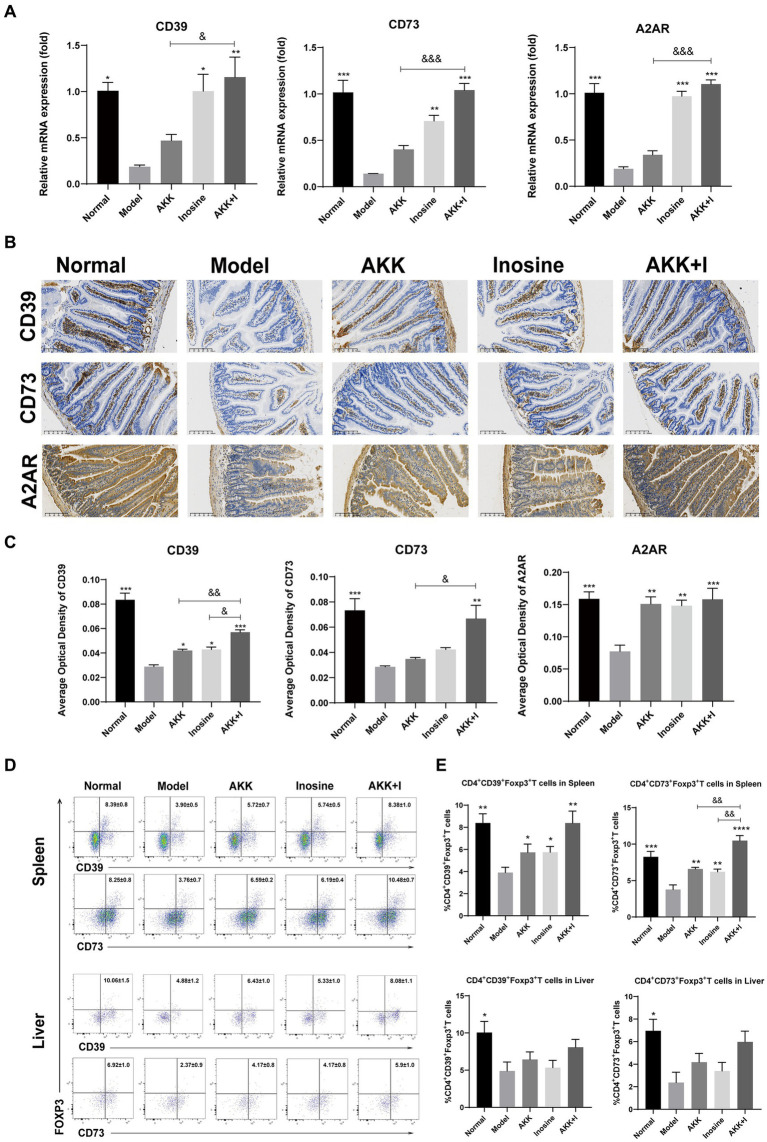
The combination of *Akkermansia muciniphila* and inosine increases the expression of CD39, CD73, and A2AR in the intestine and upregulates the ratios of CD39^+^ and CD73^+^ Treg cells in CD4^+^ T cells from the spleen and liver. ALD mice were treated as described in Figure 1. **(A)** Fold change in mRNA levels of *CD39*, *CD73*, and *A2AR* determined using qRT-PCR. **(B)** Representative images of CD39, CD73, and A2AR proteins in the small intestine, detected via immunohistochemical staining (scale bar, 100 μm). **(C)** Mean optical densities of CD39, CD73, and A2AR. **(D,E)** Flow cytometric plots of CD39^+^ Treg cells and CD73^+^ Treg cells, and quantitative analysis of cell percentages in the spleen and liver. Data are shown as the mean ± SEM; *p < 0.05, **p < 0.01, ***p < 0.001 compared to the model group; &*p* < 0.05, &&*p* < 0.01 compared with the AKK + I group. *N* = 6 each group for RT-qPCR; *N* = 10 each group for immunohistochemical staining; *N* = 7–10 each group for Flow cytometric.

The adenosine/A2AR pathway is crucial for the suppression of inflammation and immune cell differentiation. Alcohol consumption significantly reduced *CD39*, *CD73*, and *A2AR* mRNA expression levels in the small intestine compared to that in the normal group, whereas inosine administration significantly increased them (Figure 6A). In addition, *A. muciniphila*-treated mice exhibited elevated levels of these markers, but the differences were not significant compared to the model group. Treatment with the *A. muciniphila* and inosine combination significantly upregulated the mRNA expression of *CD39*, *CD73*, and *A2AR* (Figure 6A). To confirm our qRT-PCR results, we performed immunohistochemistry analysis and found that both *A. muciniphila* or inosine treatment alone increased CD39 and A2AR protein expression compared to that in the ALD model group (Figures 6B,C). However, combined *A. muciniphila* and inosine treatment further upregulated CD39, CD73, and A2AR protein levels (Figures 6B,C).

As CD39 and CD73, which are crucial for immunosuppression, are highly expressed in Tregs, we examined the proportions of CD4^+^CD39^+^ Tregs and CD4^+^CD73^+^ Tregs in the spleen and liver. The proportions of CD4^+^CD39^+^ Tregs and CD4^+^CD73^+^ Tregs in the spleen were significantly decreased in the model group but restored in the *A. muciniphila* or inosine groups, and this effect was further pronounced in the combination group (Figures 6D,E). The ratio of CD4^+^CD39^+^ Tregs to CD4^+^CD73^+^ Tregs in the liver was lower than that in the normal group, with no significant differences among the treatment groups (Figures 6D,E).

### 3.8 Therapeutic effects of *Akkermansia muciniphila* + inosine are blocked by the A2AR antagonist KW6002

**Figure 7:**
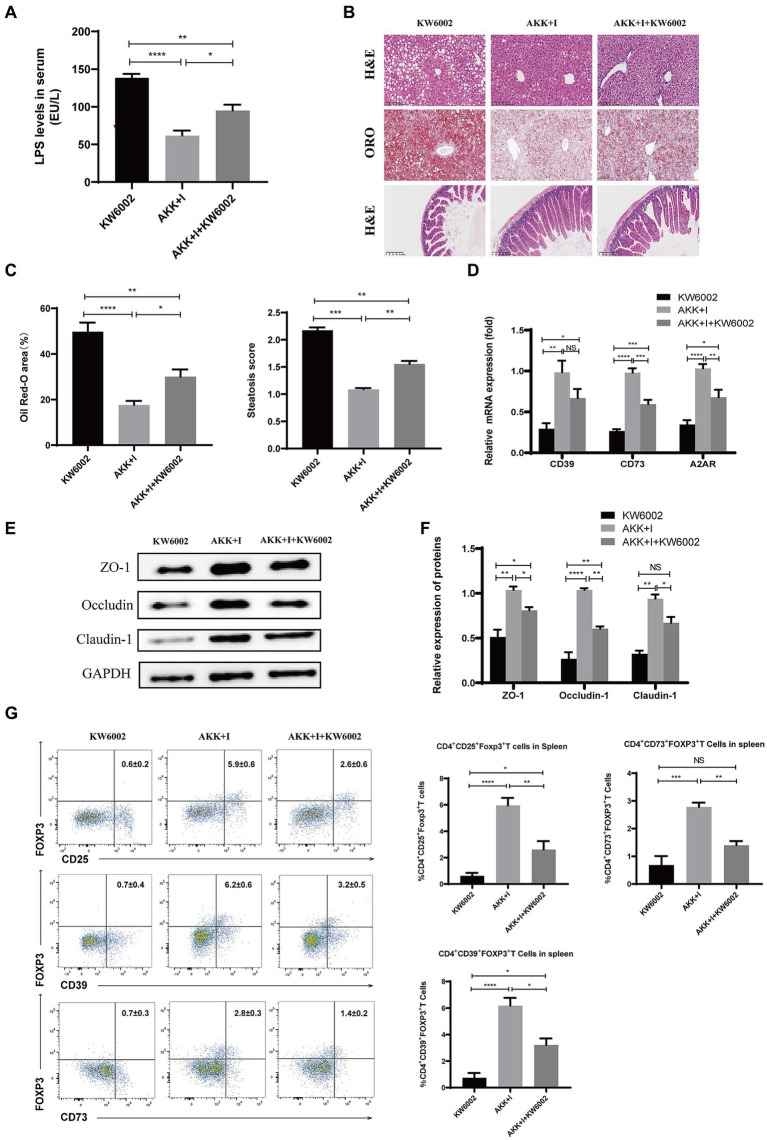
The combination of *Akkermansia muciniphila* and inosine exerts protective effects against ALD partly through A2AR. C57BL/6 mice were fed a Lieber-DeCarli diet containing 5% alcohol for 4 weeks. A combination of *A. muciniphila* (1 × 10^9^ CFU/mouse) and inosine (300 mg/kg) was administered orally every other day. KW6002 (5 mg/kg/day) was injected intraperitoneally during the last 4 weeks of the complete ethanol diet. All mice were euthanized in the 6th week. **(A)** The levels of LPS in serum. **(B)** Representative images of H&E and Oil Red O staining of liver sections (scale bar, 100 μm), and H&E-stained histological sections of the small intestine (scale bar: 200 μm). **(C)** Statistical analysis of steatosis scores and Oil Red O staining. **(D)** Fold change in mRNA levels of *CD39*, *CD73*, and *A2AR* determined via qRT-PCR. **(E,F)** Representative Western blot images and histograms of the band densities of ZO-1, Occludin, and Claudin-1 in the small intestine of each experimental group. **(G)** Flow cytometric plots of Tregs, CD39^+^ Treg, and CD73^+^ Tregs, and quantitative analysis of cell percentages in the spleen. Data are shown as the mean ± SEM; *p < 0.05, **p < 0.01, ***p < 0.001, ****p < 0.0001. *N* = 5 for each group.

We investigated whether A2AR acts as a mediator for the beneficial effects induced by the combination of *A. muciniphila* and inosine. Compared to the *A. muciniphila* combined with inosine group, the serum LPS levels in the *A. muciniphila* + inosine plus KW6002 group were significantly increased (Figure 7A), more severe balloon-like changes and fat deposition were observed in the liver (Figures 7B,C), the villus-crypt structure of the small intestine was more severely damaged (Figure 7B), and the levels of intestinal TJ proteins were significantly decreased (Figures 7E,F). In addition, compared to the *A. muciniphila* combined with inosine group, the mRNA levels of *CD39*, *CD73*, and *A2AR* in the small intestine of mice in the *A. muciniphila* + inosine plus KW6002 group were significantly reduced (Figure 7D), the proportions of Tregs, CD39^+^Tregs and CD73^+^Tregs in the spleen and liver were also significantly reduced (Figure 7G; Supplementary Figure S2). KW6002 blocked the therapeutic effect of AKK combined with inosine. Compared to the *A. muciniphila* + inosine plus KW6002 group, when KW6002 was treated alone, the mice showed the more severe pathological manifestations about these indicators. Our results suggest that the protective effect of *A. muciniphila* combined with inosine for ALD is achieved, in part, through the A2AR signaling pathway.

## 4 Discussion

ALD is the primary cause of chronic liver disease, and its end stages are associated with higher complication rates of spontaneous peritonitis and hepatic encephalopathy than other liver diseases (Bhandari et al., 2020). Research indicates that a compromised mucosal barrier and severe dysbiosis of the intestinal microbiota are factors in the immunological imbalance in the gut–liver axis that causes intrahepatic inflammation in ALD (Yan et al., 2023). Similarly, in the present study, we discovered that alcohol exposure severely damaged the intestinal barrier, resulting in the depletion of potential probiotic genera, such as *Lactobacillus*, *Clostridium* IV, and *Akkermansia*, and the enrichment of opportunistic pathogens, such as *Oscillibacter*, *Escherichia*/*Shigella*, and *Alistipes*, in ALD. Therefore, targeting the gut microbiota is a promising therapeutic strategy for treating ALD.

The abundance of *A. muciniphila* is significantly reduced in patients with ALD (Addolorato et al., 2020b). While accumulating evidence supports the beneficial role of *A. muciniphila* in autoimmune hepatitis and non-alcoholic fatty liver disease (NAFD) (Wu et al., 2017; Qu et al., 2023), its exploration in the context of ALD is limited. In a previous study, we treated ALD with varying concentrations of inosine combined with *Lactobacillus rhamnosus* and found that 300 mg/kg inosine combined with *L. rhamnosus* demonstrated superior regulation of ALD-induced intestinal microecological disorders (Zhu et al., 2022). In the present study, the combination of *A. muciniphila* and inosine alleviated liver injury, as evidenced by the reduced expression of TNF-α, IL-6, and IL-1β; alleviated hepatocyte degeneration and liver fat accumulation; reduced infiltration of macrophages and neutrophils; and corrected the redox imbalance. In addition, the combined therapy reduced serum LPS levels, downregulated hepatic TLR4, MyD88, and NF-κB expression, and upregulated intestinal TJ protein expression levels, suggesting that its anti-inflammatory effects are closely related to the improved intestinal barrier function. To the best of our knowledge, this is the first report on the efficacy of the combination of *A. muciniphila* and inosine in preventing ALD.

In this study, the combined supplementation of *A. muciniphila* and inosine significantly decreased the relative abundances of *Oscillibacter*, *Escherichia*/*Shigella*, and *Alistipes*. Prior research has reported the enrichment of *Oscillibacter* in patients with chronic metabolic diseases such as NAFLD (Rodriguez-Diaz et al., 2022). *Escherichia*/*Shigella* increases endogenous ethanol, secondary bile acids, and endotoxin production, all of which are known to exacerbate hepatic inflammation (Baltazar-Díaz et al., 2022). Alcohol consumption increases the levels of *Alistipes*, an aggravating factor associated with intestinal inflammation (Pisani et al., 2022). In contrast, the combination treatment effectively increased the relative abundance of *Lactobacillus*, *Clostridium* IV, and *Akkermansia*, three important intestinal tract producers of SCFAs (Li et al., 2023). SCFAs, which are crucial energy sources for colon epithelial cells, play a vital role in maintaining gut barrier functions by inducing genes encoding TJs that exhibit immunomodulatory and anti-inflammatory properties (Silva et al., 2018; Gonzalez et al., 2019). Higher butyrate levels decrease intestinal chemotaxis and inflammation by promoting Treg cell differentiation by inhibiting histone deacetylase or activation of GPR signaling (Arpaia et al., 2013). In addition, butyrate reduces autoimmune responses and steatohepatitis by modulating T cell development through enterohepatic immunity (Hu et al., 2018). Based on targeted metabolomic analysis, we observed that the levels of SCFAs, including propionic, butyric, valeric, and acetic acids, markedly decreased after alcohol exposure. However, the reshaping of the microbial community by *A. muciniphila* combined with inosine was characterized by the dominance of beneficial bacteria favoring SCFA synthesis. Consequently, this combination restored acetate, propionate, and butyrate levels to appropriate concentrations. These findings suggest that an increase in the abundance of SCFA-producing bacteria may play a crucial role in the regulation of the intestinal mucosal immune response.

In addition to the gut microbiota, A2AR is also involved in maintaining intestinal homeostasis (Sun et al., 2021). Studies have indicated high expression of CD39 and CD73 in immune cells within the intestine, closely related to the intestinal lumen environment (Weinhage et al., 2015; Zhu et al., 2021). Moreover, acute *Toxoplasma gondii* infection leads to a marked decrease in CD73 expression and adenosine content in the intestinal lumen, resulting in intestinal mucosal injury and exacerbation of hepatic damage, whereas activation of adenosine A2AR significantly ameliorates intestinal mucosal injury (Francois et al., 2015). In the present study, alcohol consumption reduced the expression of A2AR, CD39, and CD73 in the small intestine. Conversely, the combination of *A. muciniphila* and inosine significantly increased intestinal CD39, CD73, and A2AR expression levels in intestinal submucosal immune cells, potentially contributing to the protective effect of this symbiotic combination on intestinal immune homeostasis and the intestinal barrier.

A2AR is highly expressed on T cell surfaces and is closely associated with their differentiation and functional changes (Vigano et al., 2019). Adenosine inhibits effector T cell function by binding to A2AR, interfering with TCR activation and the downstream signaling of co-stimulatory molecules (Alam et al., 2020). In patients with alcohol-related cirrhosis, a strong correlation was observed between the severity of their condition and the Th1 response intensity, and the secretion of IFN-γ by Th1 cells directly contributed to hepatic injury (González-Reimers et al., 2012). Moreover, patients with alcoholic hepatitis exhibit liver infiltration by Th17 cells, which correlates with hepatic damage (He et al., 2021). Our study identified a significant immune cell imbalance in the alcoholic liver model group compared to the normal group. We verified that alcohol exposure increased the number of Th1 and Th17 cells, and this increase was reversed by the combination of inosine and *A. muciniphila.* Moreover, the combination therapy significantly decreased the liver expression of IL-17A and INF-γ induced by alcohol. Tregs are a subset of immune-suppressive cells that are crucial in several liver diseases (Qu et al., 2022). In the present study, alcohol reduced the proportion of Treg cells, whereas treatment with *A. muciniphila*, alone or in combination with inosine, increased the proportion of Treg cells in ALD mice. In addition, we observed a reduced number of Tregs expressing CD39 and CD73 following alcohol intake. In the context of ALD induction using CD39-knockout mice, the absence of CD39 expression in Tregs results in the impairment of their immunosuppressive capabilities. Consequently, this leads to a heightened inflammatory response in CD39-knockout mice compared with wild-type mice (Xia et al., 2022). Similarly, Tregs derived from an autoimmune hepatitis model generated a reduced amount of anti-inflammatory substances (TGF-β and IL-10) compared to control Tregs, associated with impaired CD73 expression on the Treg surface (Huang et al., 2021). Furthermore, adenosine produced by Tregs can act on A2AR on the surface of various immune cells, such as Th1, Th2, and Th17 cells. This interaction reduces the secretion of cytokines, such as IFN-γ, IL-17, and IL-2, thereby inhibiting the differentiation and maturation of Th1 and Th2 (Zhang et al., 2023). Notably, supplementation with *A. muciniphila* combined with inosine increased the proportion of CD39^+^ and CD73^+^ Tregs in the liver and spleen, suggesting that *A. muciniphila* and inosine may regulate Treg cell function through the CD39-CD73-A2AR pathway. Importantly, in this study, A2AR antagonist blocked the beneficial effects of *A. muciniphila* combined with inosine in alleviating liver injury, repairing intestinal barrier function, and increasing Treg cells proportion in ALD, suggesting that A2AR may be a potential new target for the treatment of ALD.

Regarding the future directions of our study, although supplementation with *A. muciniphila* and inosine has demonstrated promising therapeutic effects in models of ALD, the specific components of *A. muciniphila* that synergizes with inosine remains unclear. Further investigation into the interactions between *A. muciniphila*’s outer membrane proteins or vesicles and inosine is warranted. Furthermore, the specific mechanism by which *A. muciniphila* combined with inosine regulates the CD39-CD73-A2AR pathway needs to be elucidated. We plan to address this gap through future experiments using knockout mouse models and *in vitro* studies. Finally, our intervention was conducted entirely in the mouse model, and the beneficial effects from those in humans warrant further investigation in clinical trials.

## 5 Conclusion

In conclusion, this study investigated the effects of the combination of *A. muciniphila* and inosine on the gut–liver axis in alcohol-fed mice. Our results suggest that this combined treatment can ameliorate liver injury in an ALD mouse model by restoring gut microbiota balance, regulating intestinal barrier function, reducing inflammatory cytokine levels, ameliorating oxidative stress, and increasing the proportions of Tregs while decreasing the proportions of Th1 and Th17 cells. In addition, *A. muciniphila* and inosine combination therapy increased the expression of A2AR, CD73, and CD39 in the intestinal mucosa, and increased the proportions of CD39^+^ Treg cells and CD73^+^ Treg cells. This modulation contributes to the regulation of the immunity of the “gut–liver axis” and inhibits the immune response in intrahepatic inflammation. Therefore, combined therapy with *A. muciniphila* and inosine holds promise as a potential therapeutic strategy for patients with ALD.

## Data availability statement

16S rRNA gene sequencing data are deposited in the NCBI Sequence Read Archive (SRA) database with accession numbers PRJNA1071471.

## Ethics statement

The animal study was approved by Ethics Committee of the Health Science Center of Ningbo University. The study was conducted in accordance with the local legislation and institutional requirements.

## Author contributions

LW: Writing – original draft, Software, Methodology, Formal analysis, Conceptualization. YP: Writing – original draft, Methodology. YuG: Writing – original draft, Software, Investigation. YZ: Writing – original draft, Data curation. HJ: Writing – original draft, Formal analysis. YiG: Writing – original draft, Resources. CL: Writing – original draft, Investigation. YW: Writing – original draft, Data curation. JL: Writing – original draft, Resources. YC: Writing – review & editing, Project administration. CK: Writing – review & editing, Visualization. LX: Writing – review & editing, Visualization, Project administration, Conceptualization.

## References

[ref1] Addolorato G. Abenavoli L. Dallio M. Federico A. Germani G. Gitto S.. (2020a). Alcohol associated liver disease 2020: a clinical practice guideline by the Italian Association for the Study of the liver (AISF). Dig. Liver Dis. 52, 374–391. doi: 10.1016/j.dld.2019.12.008, PMID: 32001151

[ref2] Addolorato G. Ponziani F. R. Dionisi T. Mosoni C. Vassallo G. A. Sestito L.. (2020b). Gut microbiota compositional and functional fingerprint in patients with alcohol use disorder and alcohol-associated liver disease. Liver Int. 40, 878–888. doi: 10.1111/liv.14383, PMID: 31951082

[ref3] Alam M. S. Cavanaugh C. Pereira M. Babu U. Williams K. (2020). Susceptibility of aging mice to listeriosis: role of anti-inflammatory responses with enhanced Treg-cell expression of CD39/CD73 and Th-17 cells. Int. J. Med. Microbiol. 310:151397. doi: 10.1016/j.ijmm.2020.151397, PMID: 31974050

[ref4] Albillos A. de Gottardi A. Rescigno M. (2020). The gut-liver axis in liver disease: pathophysiological basis for therapy. J. Hepatol. 72, 558–577. doi: 10.1016/j.jhep.2019.10.003, PMID: 31622696

[ref5] Arpaia N. Campbell C. Fan X. Dikiy S. van der Veeken J. deRoos P.. (2013). Metabolites produced by commensal bacteria promote peripheral regulatory T-cell generation. Nature 504, 451–455. doi: 10.1038/nature12726, PMID: 24226773 PMC3869884

[ref6] Avila M. A. Dufour J. F. Gerbes A. L. Zoulim F. Bataller R. Burra P.. (2020). Recent advances in alcohol-related liver disease (ALD): summary of a gut round table meeting. Gut 69, 764–780. doi: 10.1136/gutjnl-2019-319720, PMID: 31879281 PMC7236084

[ref7] Baltazar-Díaz T. A. González-Hernández L. A. Aldana-Ledesma J. M. Peña-Rodríguez M. Vega-Magaña A. N. Zepeda-Morales A. S. M.. (2022). Escherichia/shigella, SCFAs, and metabolic pathways-the triad that orchestrates intestinal dysbiosis in patients with decompensated alcoholic cirrhosis from Western Mexico. Microorganisms 10:1231. doi: 10.3390/microorganisms10061231, PMID: 35744749 PMC9229093

[ref8] Bhandari R. Khaliq K. Ravat V. Kaur P. Patel R. S. (2020). Chronic alcoholic liver disease and mortality risk in spontaneous bacterial peritonitis: analysis of 6,530 hospitalizations. Cureus 12:e8189. doi: 10.7759/cureus.8189, PMID: 32566430 PMC7301415

[ref9] Cabezas J. (2022). Management of Alcohol-Related Liver Disease and its Complications. Clin. Drug Investig. 42, 47–53. doi: 10.1007/s40261-022-01143-9, PMID: 35467296 PMC9205805

[ref10] Cani P. D. Depommier C. Derrien M. Everard A. de Vos W. M. (2022). *Akkermansia muciniphila*: paradigm for next-generation beneficial microorganisms. Nat. Rev. Gastroenterol. Hepatol. 19, 625–637. doi: 10.1038/s41575-022-00631-9 35641786

[ref11] Cani P. D. Van Hul M. Bachmann R. (2023). *Akkermansia muciniphila* derived tripeptide jams the gear of sepsis, inflammation and mortality. Gut 73, 3–4. doi: 10.1136/gutjnl-2023-331092, PMID: 37857477

[ref12] Francois V. Shehade H. Acolty V. Preyat N. Delrée P. Moser M.. (2015). Intestinal immunopathology is associated with decreased CD73-generated adenosine during lethal infection. Mucosal Immunol. 8, 773–784. doi: 10.1038/mi.2014.108, PMID: 25389034

[ref13] Gao B. Bataller R. (2011). Alcoholic liver disease: pathogenesis and new therapeutic targets. Gastroenterology 141, 1572–1585. doi: 10.1053/j.gastro.2011.09.002, PMID: 21920463 PMC3214974

[ref14] Ge L. Chen D. Chen W. Cai C. Tao Y. Ye S.. (2019). Pre-activation of TLR3 enhances the therapeutic effect of BMMSCs through regulation the intestinal HIF-2α signaling pathway and balance of NKB cells in experimental alcoholic liver injury. Int. Immunopharmacol. 70, 477–485. doi: 10.1016/j.intimp.2019.02.021, PMID: 30870678

[ref15] Ge Y. Sun H. Xu L. Zhang W. Lv J. Chen Y. (2022). The amelioration of alcohol-induced liver and intestinal barrier injury by *Lactobacillus rhamnosus* Gorbach-Goldin (LGG) is dependent on interleukin 22 (IL-22) expression. Bioengineered 13, 12650–12660. doi: 10.1080/21655979.2022.2070998, PMID: 35603884 PMC9275995

[ref16] Gonzalez A. Krieg R. Massey H. D. Carl D. Ghosh S. Gehr T. W. B.. (2019). Sodium butyrate ameliorates insulin resistance and renal failure in CKD rats by modulating intestinal permeability and mucin expression. Nephrol. Dial. Transplant. 34, 783–794. doi: 10.1093/ndt/gfy238, PMID: 30085297 PMC6503301

[ref17] González-Reimers E. Santolaria-Fernández F. Medina-García J. A. González-Pérez J. M. de la Vega-Prieto M. J. Medina-Vega L.. (2012). TH-1 and TH-2 cytokines in stable chronic alcoholics. Alcohol Alcohol 47, 390–396. doi: 10.1093/alcalc/ags041, PMID: 22510812

[ref18] Grander C. Adolph T. E. Wieser V. Lowe P. Wrzosek L. Gyongyosi B.. (2018). Recovery of ethanol-induced *Akkermansia muciniphila* depletion ameliorates alcoholic liver disease. Gut 67, 891–901. doi: 10.1136/gutjnl-2016-313432, PMID: 28550049

[ref19] Guo W. Xiang Q. Mao B. Tang X. Cui S. Li X.. (2021). Protective effects of microbiome-derived inosine on lipopolysaccharide-induced acute liver damage and inflammation in mice via mediating the TLR4/NF-κB pathway. J. Agric. Food Chem. 69, 7619–7628. doi: 10.1021/acs.jafc.1c01781, PMID: 34156842

[ref20] Haskó G. Linden J. Cronstein B. Pacher P. (2008). Adenosine receptors: therapeutic aspects for inflammatory and immune diseases. Nat. Rev. Drug Discov. 7, 759–770. doi: 10.1038/nrd2638 18758473 PMC2568887

[ref21] He Y. Hwang S. Ahmed Y. A. Feng D. Li N. Ribeiro M.. (2021). Immunopathobiology and therapeutic targets related to cytokines in liver diseases. Cell. Mol. Immunol. 18, 18–37. doi: 10.1038/s41423-020-00580-w, PMID: 33203939 PMC7853124

[ref22] Hu E. D. Chen D. Z. Wu J. L. Lu F. B. Chen L. Zheng M. H.. (2018). High fiber dietary and sodium butyrate attenuate experimental autoimmune hepatitis through regulation of immune regulatory cells and intestinal barrier. Cell. Immunol. 328, 24–32. doi: 10.1016/j.cellimm.2018.03.003, PMID: 29627063

[ref23] Huang D. Q. Mathurin P. Cortez-Pinto H. Loomba R. (2023). Global epidemiology of alcohol-associated cirrhosis and HCC: trends, projections and risk factors. Nat. Rev. Gastroenterol. Hepatol. 20, 37–49. doi: 10.1038/s41575-022-00688-6, PMID: 36258033 PMC9579565

[ref24] Huang C. Shen Y. Shen M. Fan X. Men R. Ye T.. (2021). Glucose metabolism reprogramming of regulatory T cells in concanavalin A-induced hepatitis. Front. Pharmacol. 12:726128. doi: 10.3389/fphar.2021.726128, PMID: 34531750 PMC8438122

[ref25] Li N. Wang H. Zhao H. Wang M. Cai J. Hao Y.. (2023). Cooperative interactions between Veillonella ratti and *Lactobacillus acidophilus* ameliorate DSS-induced ulcerative colitis in mice. Food Funct. 14, 10475–10492. doi: 10.1039/d3fo03898j, PMID: 37934670

[ref26] Liu Y. Yang M. Tang L. Wang F. Huang S. Liu S.. (2022). TLR4 regulates RORγt(+) regulatory T-cell responses and susceptibility to colon inflammation through interaction with *Akkermansia muciniphila*. Microbiome 10:98. doi: 10.1186/s40168-022-01296-x, PMID: 35761415 PMC9235089

[ref27] Nowak M. Lynch L. Yue S. Ohta A. Sitkovsky M. Balk S. P.. (2010). The A2aR adenosine receptor controls cytokine production in iNKT cells. Eur. J. Immunol. 40, 682–687. doi: 10.1002/eji.200939897, PMID: 20039304 PMC2967447

[ref28] Odashima M. Bamias G. Rivera-Nieves J. Linden J. Nast C. C. Moskaluk C. A.. (2005). Activation of A2A adenosine receptor attenuates intestinal inflammation in animal models of inflammatory bowel disease. Gastroenterology 129, 26–33. doi: 10.1053/j.gastro.2005.05.032, PMID: 16012931

[ref29] Pisani A. Rausch P. Bang C. Ellul S. Tabone T. Marantidis Cordina C.. (2022). Dysbiosis in the gut microbiota in patients with inflammatory bowel disease during remission. Microbiol. Spect. 10:e0061622. doi: 10.1128/spectrum.00616-22, PMID: 35532243 PMC9241752

[ref30] Qu G. Chen J. Li Y. Yuan Y. Liang R. Li B. (2022). Current status and perspectives of regulatory T cell-based therapy. J. Genet. Genomics 49, 599–611. doi: 10.1016/j.jgg.2022.05.005, PMID: 35636740

[ref31] Qu D. Chen M. Zhu H. Liu X. Cui Y. Zhou W.. (2023). Akkermansia muciniphila and its outer membrane protein Amuc_1100 prevent high-fat diet-induced nonalcoholic fatty liver disease in mice. Biochem. Biophys. Res. Commun. 684:149131. doi: 10.1016/j.bbrc.2023.149131, PMID: 37866242

[ref32] Rodrigues V. F. Elias-Oliveira J. Pereira Í. Pereira J. A. Barbosa S. C. Machado M. S. G.. (2022). Akkermansia muciniphila and gut immune system: a good friendship that attenuates inflammatory bowel disease, obesity, and diabetes. Front. Immunol. 13:934695. doi: 10.3389/fimmu.2022.934695, PMID: 35874661 PMC9300896

[ref33] Rodriguez-Diaz C. Taminiau B. García-García A. Cueto A. Robles-Díaz M. Ortega-Alonso A.. (2022). Microbiota diversity in nonalcoholic fatty liver disease and in drug-induced liver injury. Pharmacol. Res. 182:106348. doi: 10.1016/j.phrs.2022.106348, PMID: 35817360

[ref34] Saveljeva S. Sewell G. W. Ramshorn K. Cader M. Z. West J. A. Clare S.. (2022). A purine metabolic checkpoint that prevents autoimmunity and autoinflammation. Cell Metab. 34, 106–124.e10. doi: 10.1016/j.cmet.2021.12.009, PMID: 34986329 PMC8730334

[ref35] Siddiqui M. T. Cresci G. A. M. (2020). Microbiota reprogramming for treatment of alcohol-related liver disease. Transl. Res. 226, 26–38. doi: 10.1016/j.trsl.2020.07.004, PMID: 32687975 PMC7572584

[ref36] Silva J. P. B. Navegantes-Lima K. C. Oliveira A. L. B. Rodrigues D. V. S. Gaspar S. L. F. Monteiro V. V. S.. (2018). Protective mechanisms of butyrate on inflammatory bowel disease. Curr. Pharm. Des. 24, 4154–4166. doi: 10.2174/1381612824666181001153605 30277149

[ref37] Sun L. Li X. Guan H. Chen S. Fan X. Zhou C.. (2021). A novel role of a(2A)R in the maintenance of intestinal barrier function of enteric glia from hypoxia-induced injury by combining with mGluR5. Front. Pharmacol. 12:633403. doi: 10.3389/fphar.2021.633403 34093180 PMC8173626

[ref38] Szabo G. (2015). Gut-liver axis in alcoholic liver disease. Gastroenterology 148, 30–36. doi: 10.1053/j.gastro.2014.10.042, PMID: 25447847 PMC4274189

[ref39] Vigano S. Alatzoglou D. Irving M. Ménétrier-Caux C. Caux C. Romero P.. (2019). Targeting adenosine in cancer immunotherapy to enhance T-cell function. Front. Immunol. 10:925. doi: 10.3389/fimmu.2019.00925 31244820 PMC6562565

[ref40] Weinhage T. Däbritz J. Brockhausen A. Wirth T. Brückner M. Belz M.. (2015). Granulocyte macrophage Colony-stimulating factor-activated CD39(+)/CD73(+) murine monocytes modulate intestinal inflammation via induction of regulatory T cells. Cell. Mol. Gastroenterol. Hepatol. 1, 433–449.e1. doi: 10.1016/j.jcmgh.2015.04.005, PMID: 28210690 PMC5301274

[ref41] Wu W. Lv L. Shi D. Ye J. Fang D. Guo F.. (2017). Protective effect of *Akkermansia muciniphila* against immune-mediated liver injury in a mouse model. Front. Microbiol. 8:1804. doi: 10.3389/fmicb.2017.01804, PMID: 29033903 PMC5626943

[ref42] Xia G. Q. Cai J. N. Wu X. Fang Q. Zhao N. Lv X. W. (2022). The mechanism by which ATP regulates alcoholic steatohepatitis through P2X4 and CD39. Eur. J. Pharmacol. 916:174729. doi: 10.1016/j.ejphar.2021.174729, PMID: 34973190

[ref43] Xiao J. Wang F. Wong N. K. He J. Zhang R. Sun R.. (2019). Global liver disease burdens and research trends: analysis from a Chinese perspective. J. Hepatol. 71, 212–221. doi: 10.1016/j.jhep.2019.03.004, PMID: 30871980

[ref44] Xing J. Zhang J. Wang J. (2023). The immune regulatory role of adenosine in the tumor microenvironment. Int. J. Mol. Sci. 24:14928. doi: 10.3390/ijms241914928, PMID: 37834375 PMC10573203

[ref45] Xu L. Wang X. Chen Y. Soong L. Chen Y. Cai J.. (2021). Metformin modulates T cell function and alleviates liver injury through bioenergetic regulation in viral hepatitis. Front. Immunol. 12:638575. doi: 10.3389/fimmu.2021.638575, PMID: 33968030 PMC8097169

[ref46] Yan C. Hu W. Tu J. Li J. Liang Q. Han S. (2023). Pathogenic mechanisms and regulatory factors involved in alcoholic liver disease. J. Transl. Med. 21:300. doi: 10.1186/s12967-023-04166-8, PMID: 37143126 PMC10158301

[ref47] Zhang C. Wang K. Wang H. (2023). Adenosine in cancer immunotherapy: taking off on a new plane. Biochim. Biophys. Acta Rev. Cancer 1878:189005. doi: 10.1016/j.bbcan.2023.189005, PMID: 37913941

[ref48] Zhu Y. Wang X. Zhu L. Tu Y. Chen W. Gong L.. (2022). *Lactobacillus rhamnosus* GG combined with inosine ameliorates alcohol-induced liver injury through regulation of intestinal barrier and Treg/Th1 cells. Toxicol. Appl. Pharmacol. 439:115923. doi: 10.1016/j.taap.2022.115923, PMID: 35176292

[ref49] Zhu Y. Zhuang Z. Wu Q. Lin S. Zhao N. Zhang Q.. (2021). CD39/CD73/A2a adenosine metabolic pathway: targets for moxibustion in treating DSS-induced ulcerative colitis. Am. J. Chin. Med. 49, 661–676. doi: 10.1142/s0192415x21500300 33683190

