# Supplementary material for: Symbiotic combination of Akkermansia muciniphila and inosine alleviates alcohol-induced liver injury by modulating gut dysbiosis and immune responses
Source: Front Microbiol. 2024 Mar 20;15:1355225. doi: 10.3389/fmicb.2024.1355225 (PMC10987824; doi:10.3389/fmicb.2024.1355225)

Supplementary materials

Supplementary Table S1 Primer pairs of Real-time Quantitative PCR analysis

Name	Sequences of primers	
CD39	Forward	GTGTATGTGTGGGTCTGTGC
	Reverse	CCAGCTTGGAAAGACTGACG
CD73	Forward	CTGAGCGCTCTACTACCACA
	Reverse	AACAGCACGTTGGGTTCTTC
A2AR	Forward	CATCCTGGGTAACGTGCTT
	Reverse	AGTGCTGATGGTGATGGC
ZO-1	Forward	GAGCAGGCTTTGGAGGAGAC
	Reverse	TGGGACAAAAGTCCGGGAAG
Occludin	Forward	CCATCTTTCTTCGGGTTT
	Reverse	TGGATCTATGTACGGCTCAC
Claudin-1	Forward	AGCGTAAAACCAGTAAGAATCCCA
	Reverse	CAAACAAGACAGCTCCAGACCAAA
IL-1β	Forward	GCCACCTTTTGACAGTGATGAG
	Reverse	ATGTGCTGCTGCGAGATTTG
IL-2	Forward	GAAACTCCCCAGGATGCTCA
	Reverse	CGCAGAGGTCCAAGTTCATCT
IL-6	Forward	GAGGATACCACTCCCAACAGACC
	Reverse	AAGTGCATCATCGTTGTTCATACA
IL-10	Forward	AGGCGCTGTCATCGATTTCT
	Reverse	ATGGCCTTGTAGACACCTTGG
TNF-α	Forward	GCGCCAAGCATTCAATGAGC
	Reverse	ATCTCTTCCCCACCCCGAAT
IFN-γ	Forward	GAGGTCAACAACCCACAGGT
	Reverse	GGGACAATCTCTTCCCCACC
TLR4	Forward	CACCAGGAAGCTTGAATCCCT
	Reverse	GGAATGTCATCAGGGACTTTGC
MyD88	Forward	GCCAGATTCTCTGATGCCGT
	Reverse	TGGGAGGAAAGGCAGTCCTA
NF-κB	Forward	TGGAGATACGGCGCTTCATC
	Reverse	AGCCAGAGTTTCCTGTGTGC
iNOS	Forward	ACGCTCGGAACTGTAGCACA
	Reverse	GCACATCAAAGCGGCCA
COX2	Forward	CAATGGGCTGGAAGACATATCAA
	Reverse	GCCAGGGCTGAACTTCGAA
AMPK	Forward	TCTGAGGGGCACCAAGAAAC
	Reverse	GTGGGTGACGGAGAAGAG
Nfr2	Forward	CCTCCGCTGCCATCAGTCAGT
	Reverse	TCGGCTGGGACTCGTGTTCA
IκB-α	Forward	AATCCTGACCTGGTTTCGCTCTT
	Reverse	ATCCTCGCTCTCGGGTAGCAT
GAPDH	Forward	AAGAAGGTGGTGAAGCAGG
	Reverse	GAAGGTGGAAGAGTGGGAGT

Figure. S1 Graphical abstract.

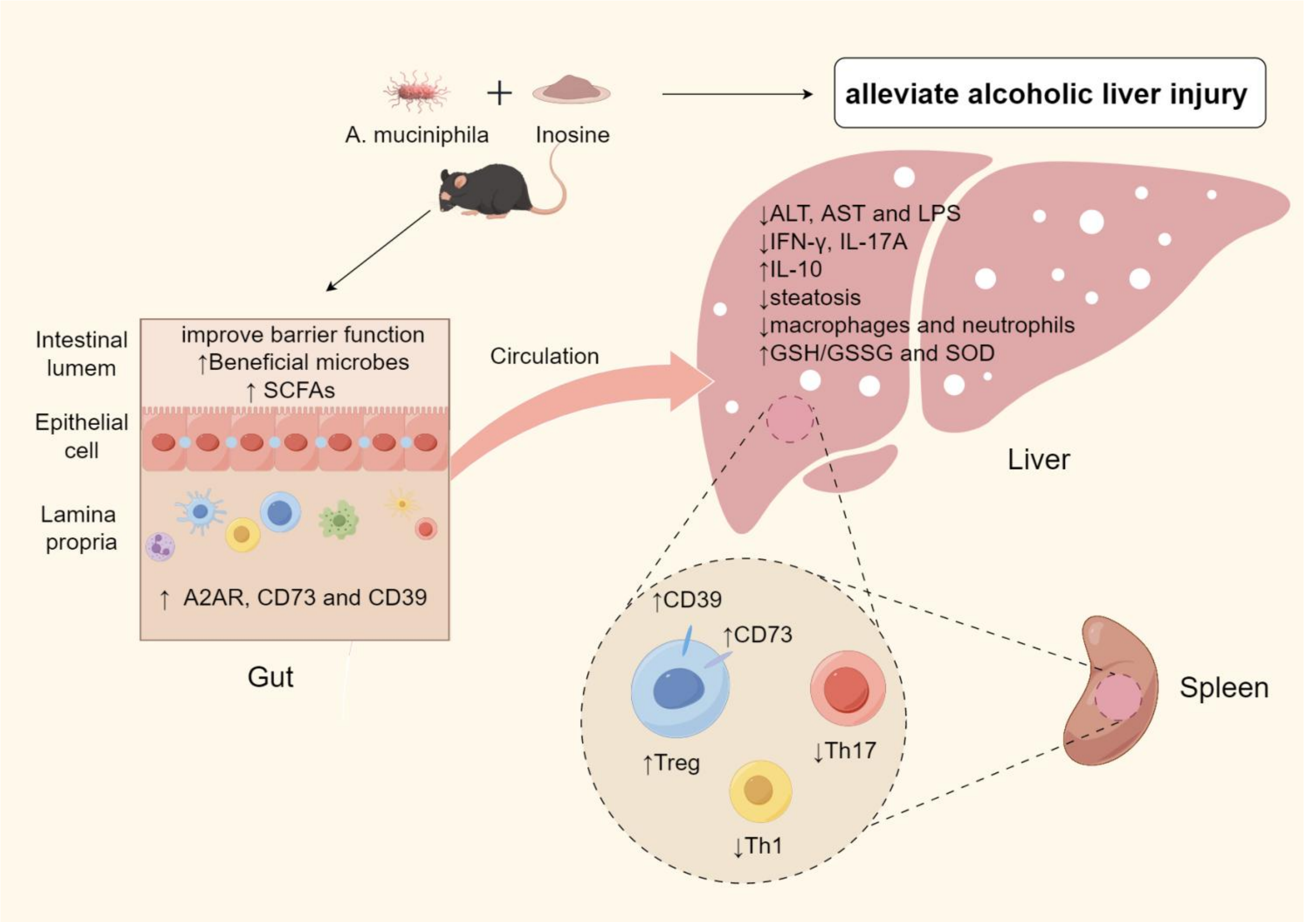

Figure. S2 Animals experimental design;

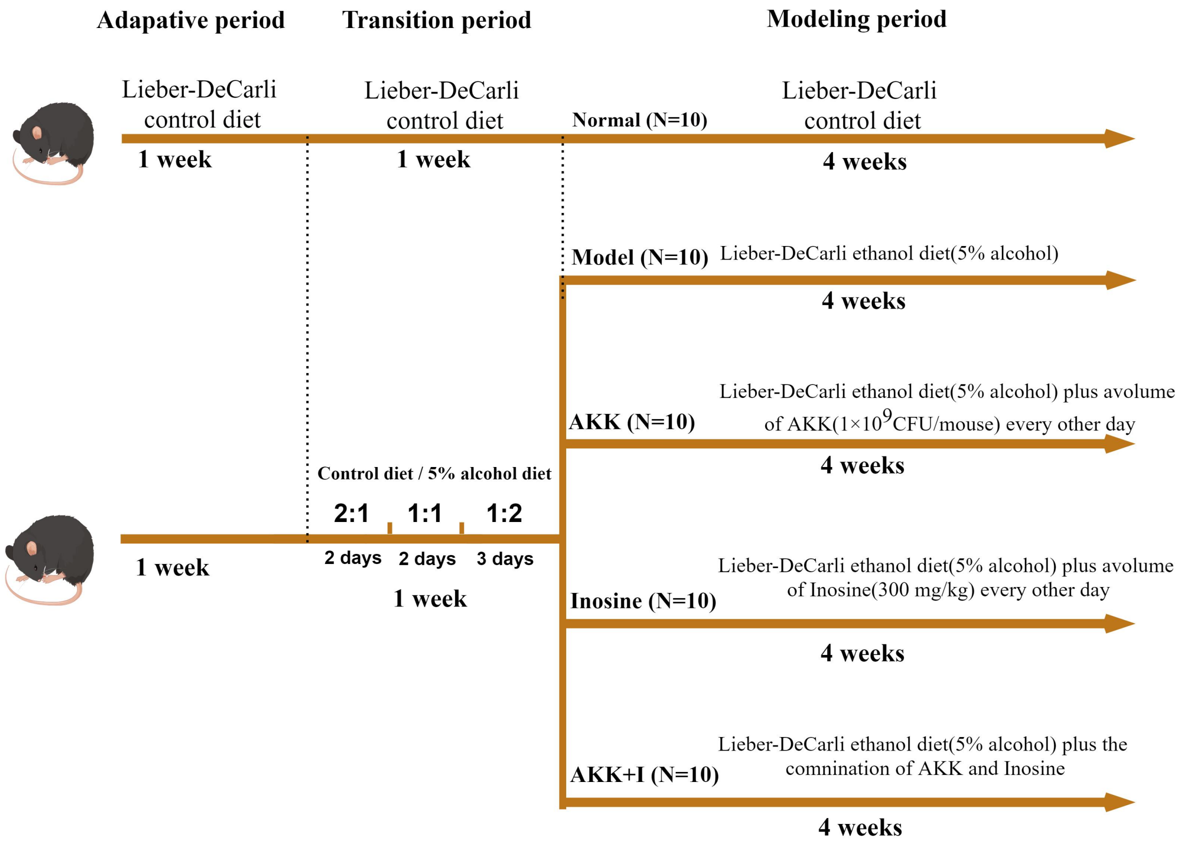

Figure. S3 ALD mice were treated as Fig. 1 described. The combined treatment reduced infiltration of macrophages and neutrophils in the liver. (A) Representative images of immunohistochemical staining for MPO and F4/80, scale bar, 100 μm. (B,C) The mean density of MPO and F4/80, respectively, representing the statistics of positive immunohistochemical staining results. Data are shown as mean ± SEM, *P < 0.05, **P < 0.01, ***P < 0.001 compared to model group; &P < 0.05, &&P < 0.01 between any 2 groups below the line.

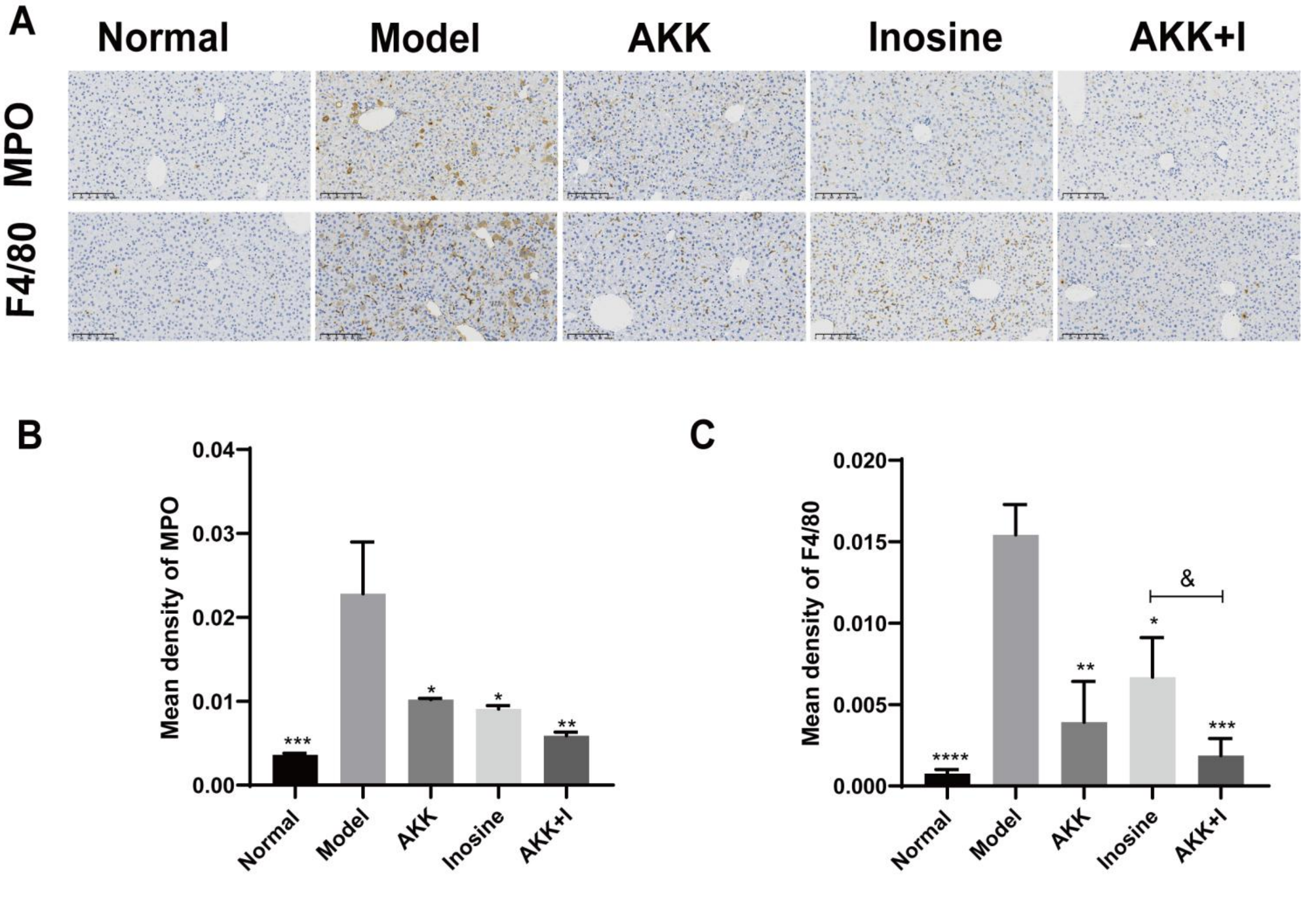

Figure. S4 ALD mice were treated as Fig. 1 described. Hepatic level of GSH (A), GSSG (B), SOD (D) and ratio of GSH/GSSG (C). Data are shown as mean ± SEM, *P < 0.05, **P < 0.01, ***P < 0.001 compared to model group; &P < 0.05, &&P < 0.01 between any 2 groups below the line.

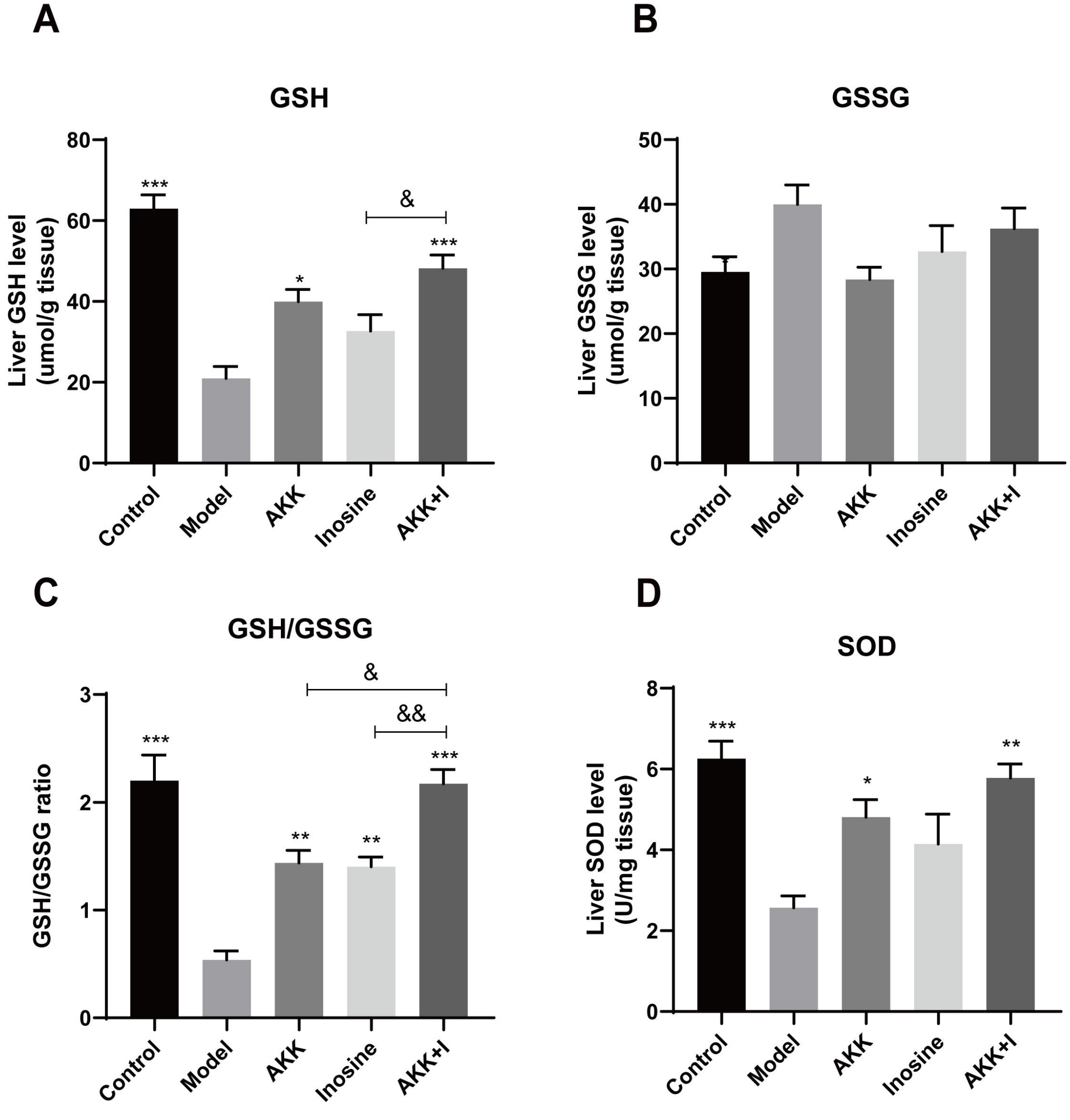

Figure. S5 ALD mice were treated as Fig. 1 described. Relative hepatic mRNA expression of IL-2 (A), IL-10 (B), IFN-γ (C), COX-2 (D), IκB-α (E), AMPK (F) and Nfr2 (G). Data are shown as mean ± SEM, *P < 0.05, **P < 0.01, ***P < 0.001 compared to model group; &P < 0.05, &&P < 0.01 between any 2 groups below the line.

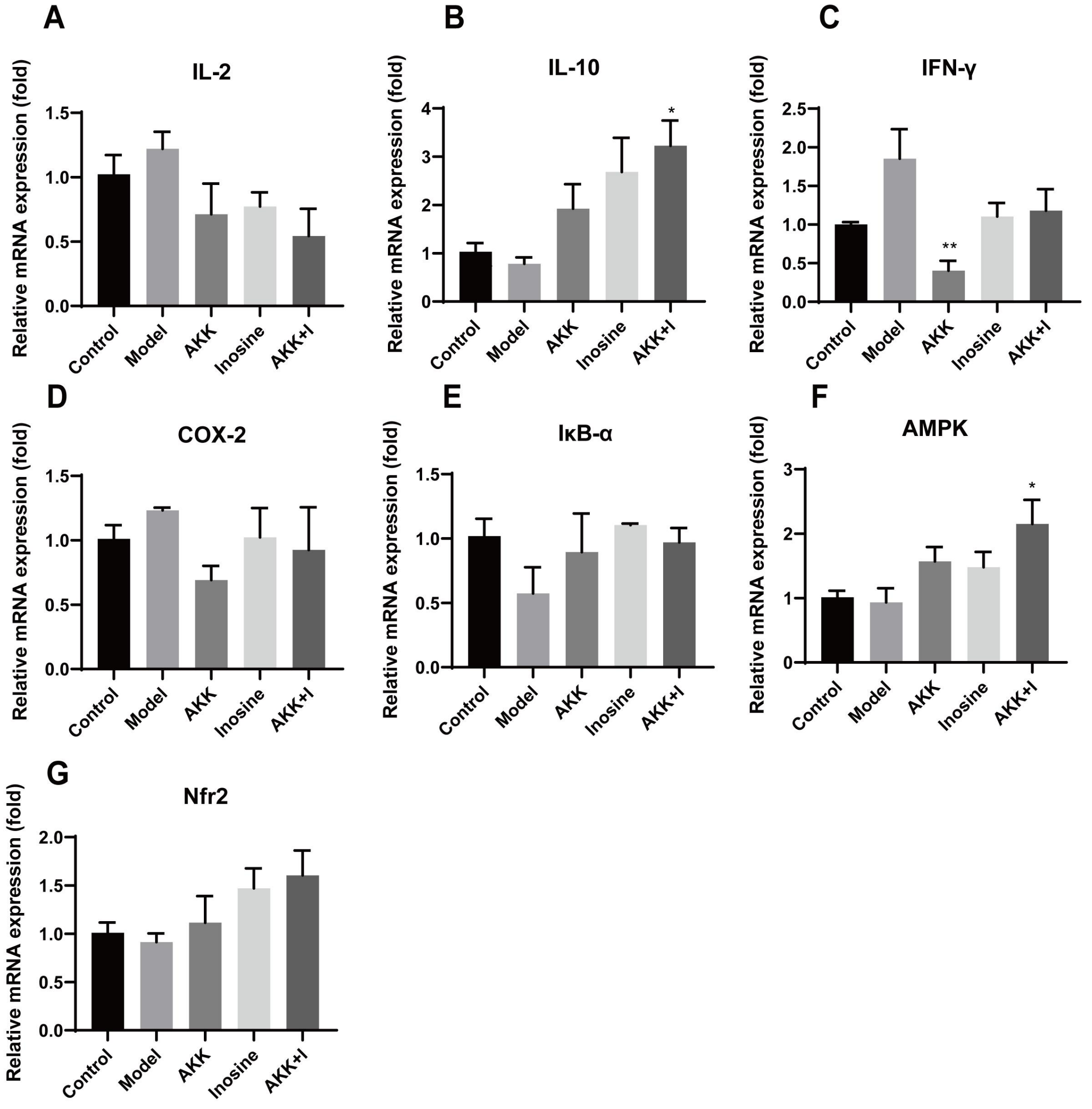

Figure. S6 The LDA histogram of significant microbial species (LDA score > 2).

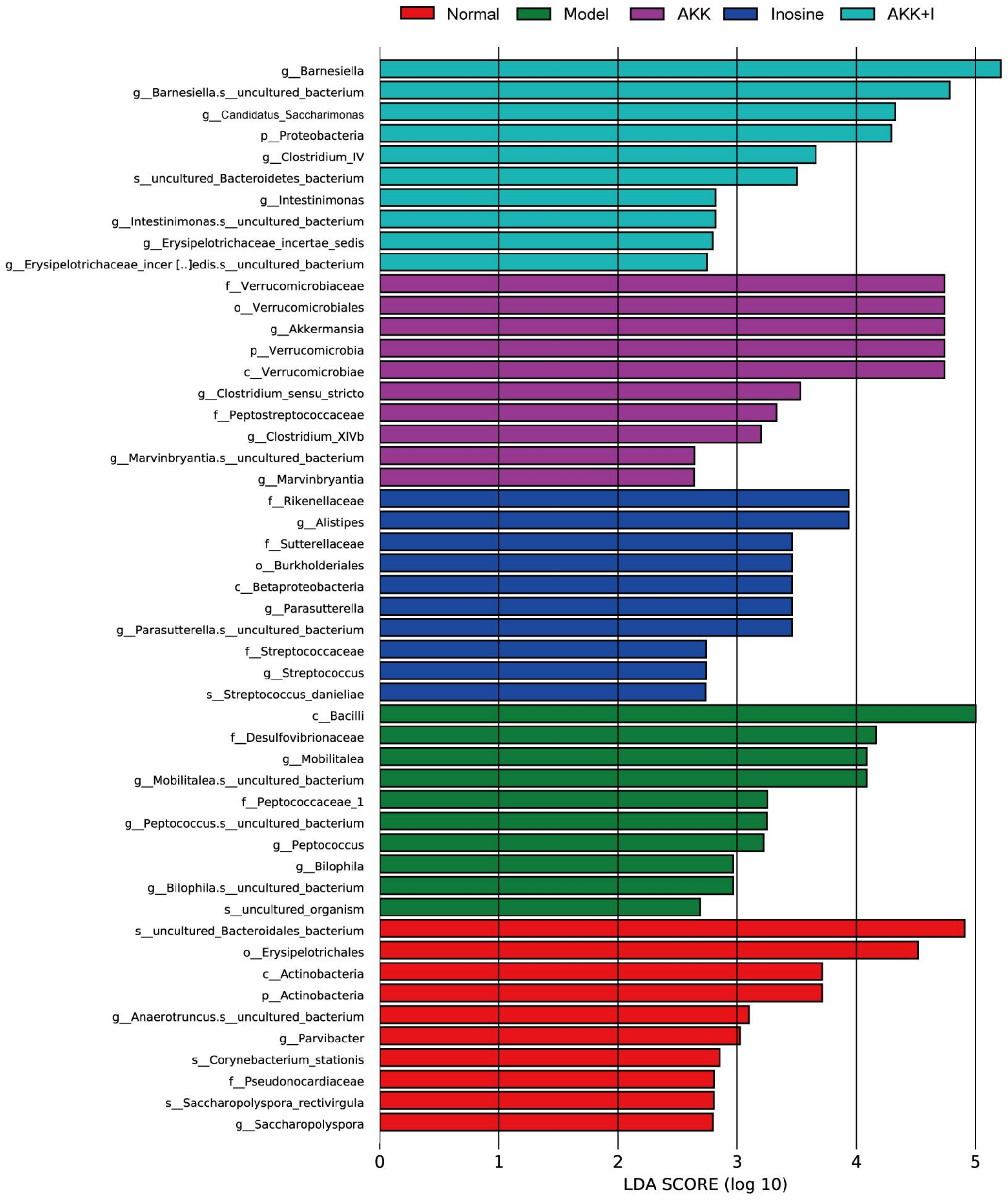

Figure. S7 Mice were treated as Fig. 7 described. (A,B) Flow cytometric plots of Tregs, CD39+ Treg cells and CD73+ Treg cells, and quantitative analysis of cell percentages in the liver. Data are shown as the mean ± SEM; *P < 0.05, **P < 0.01, ***P < 0.001.

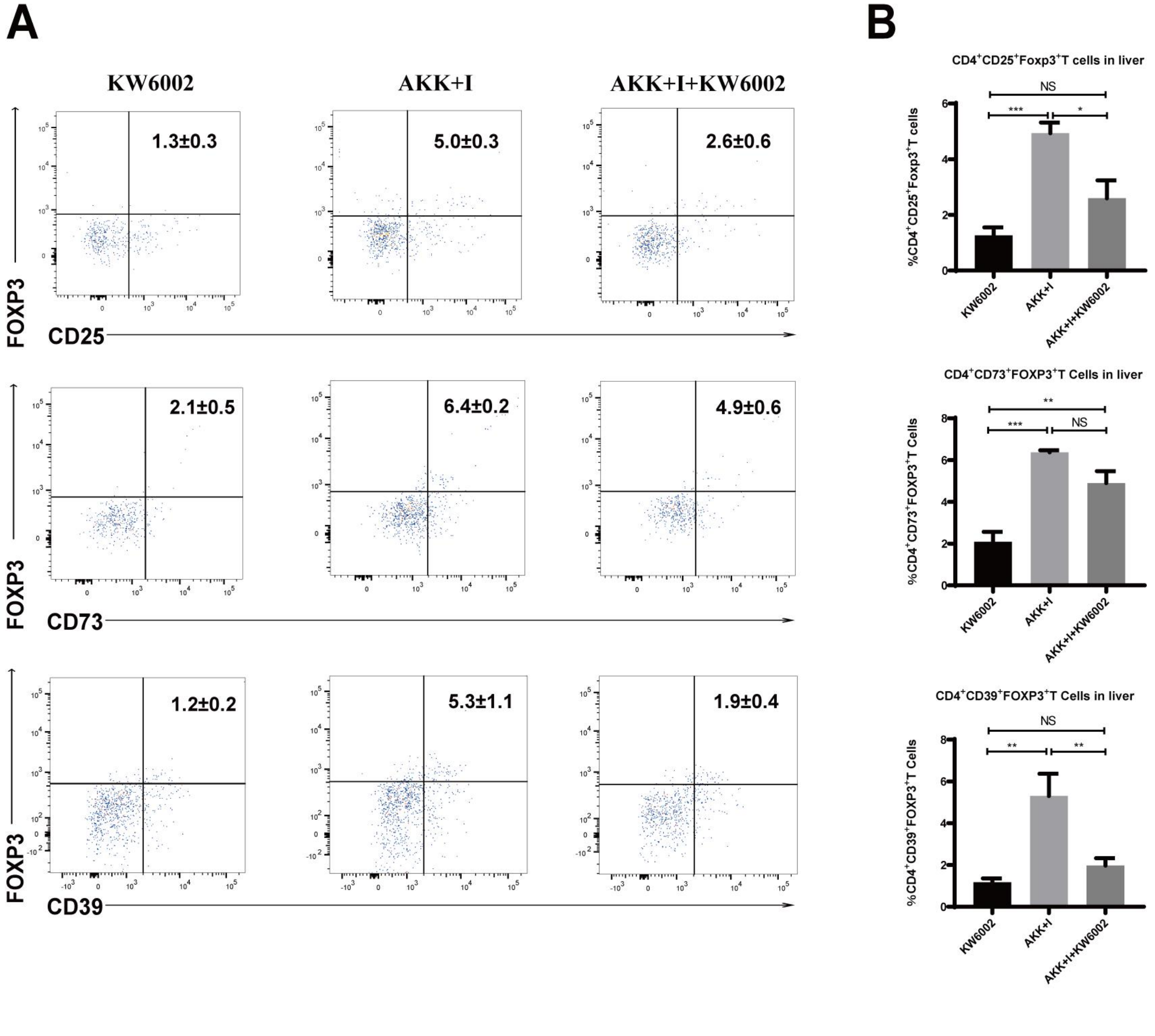

Figure. S8 Western blots Fig. 2D.

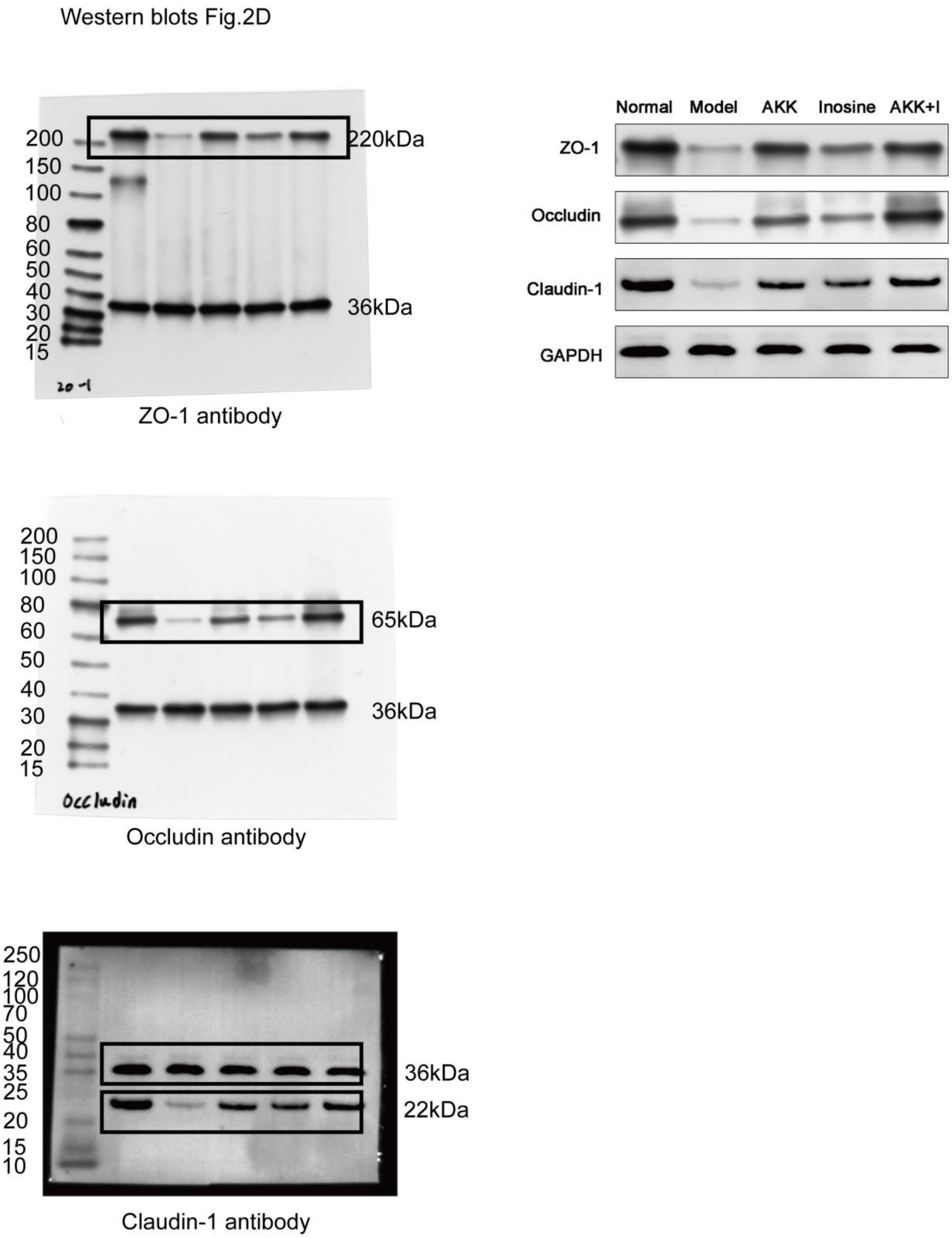

Figure. S9 Western blots Fig. 7F.

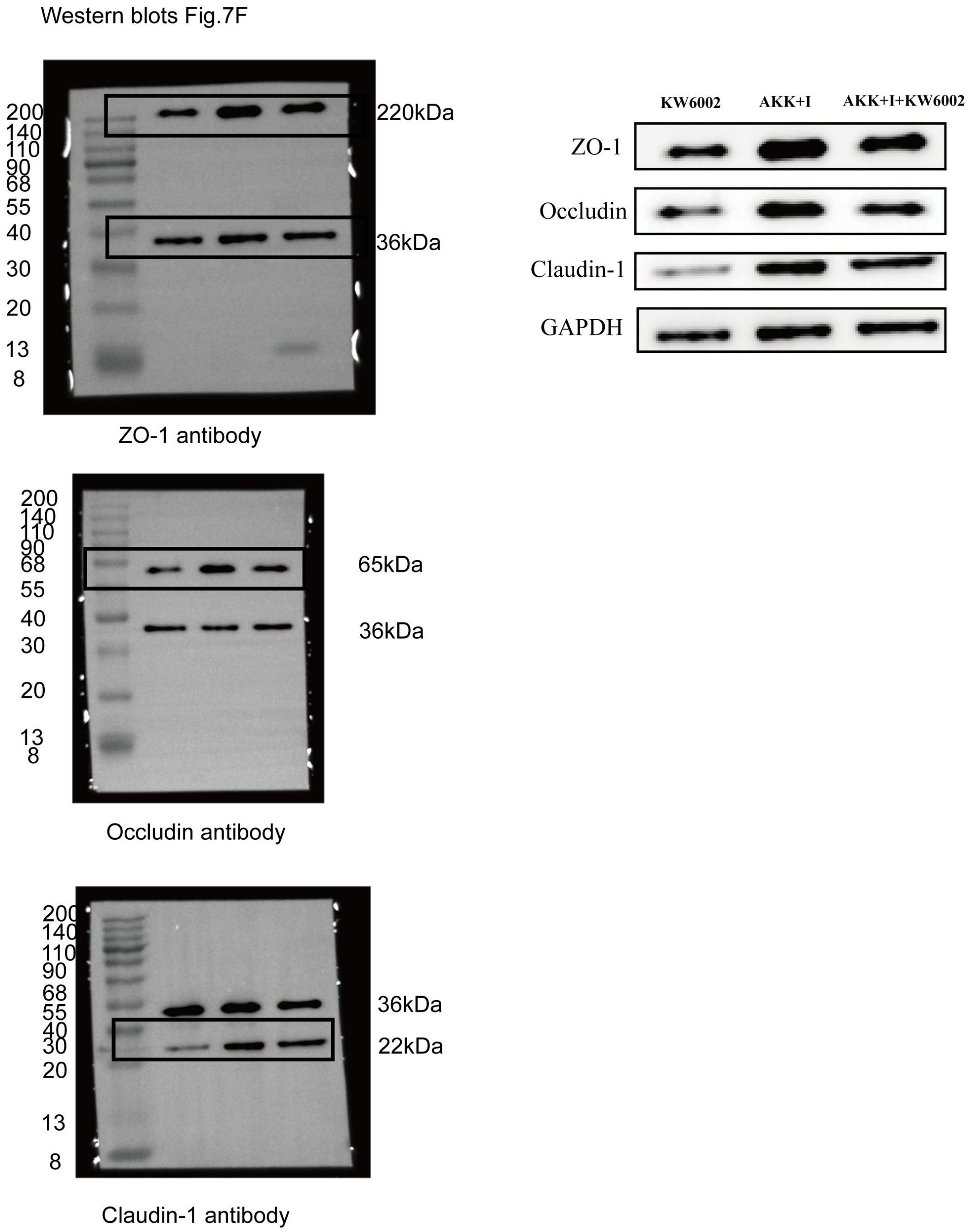

Figure. S10 The Gating strategy in flow cytometry

The Gating strategy of Tregs

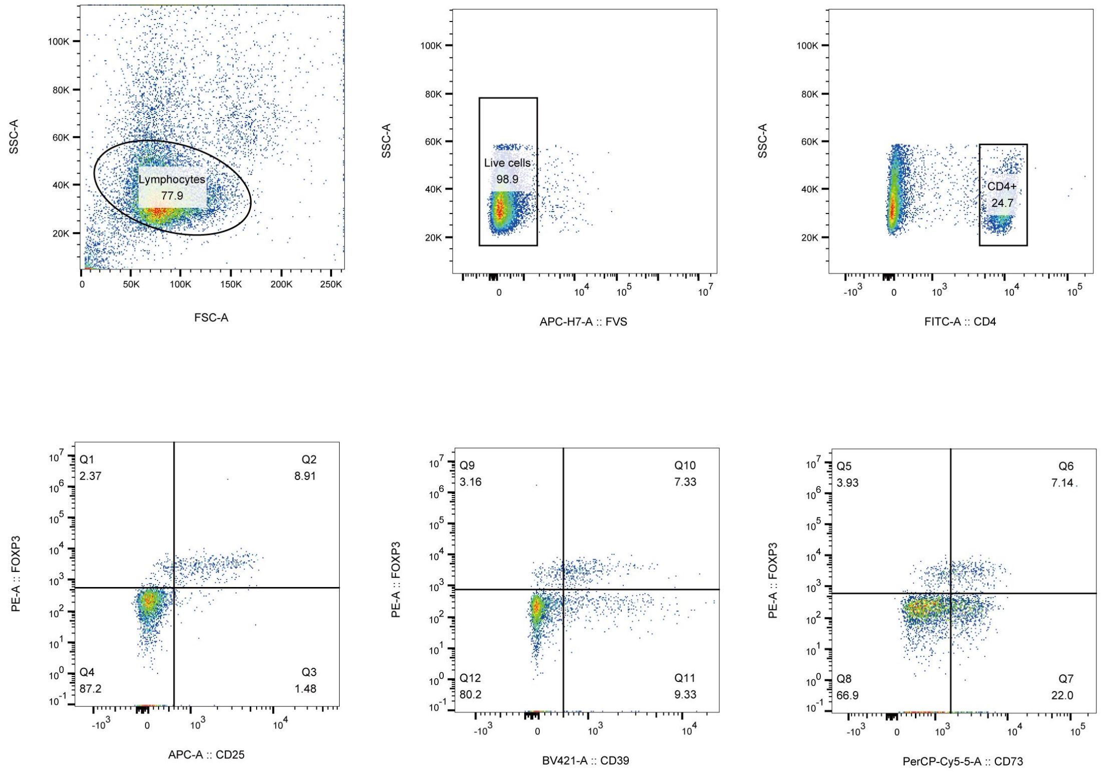

The Gating strategy of Th17 and Th1 cells

Th17 cells

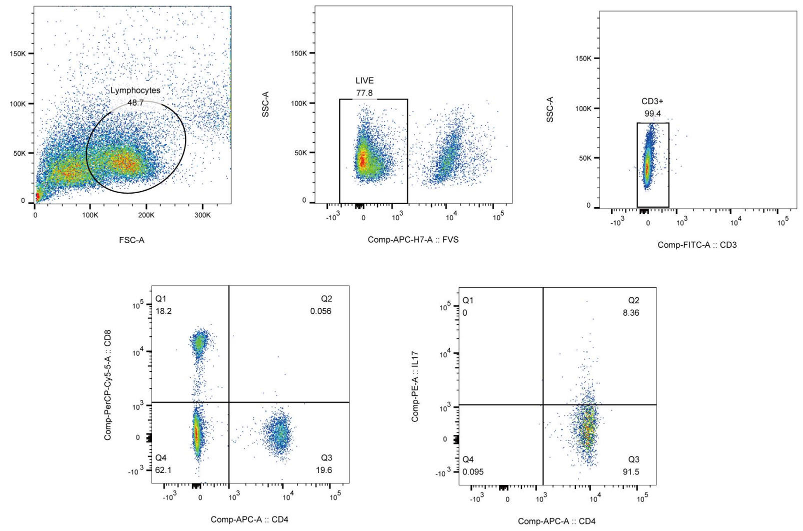

Th1 cells

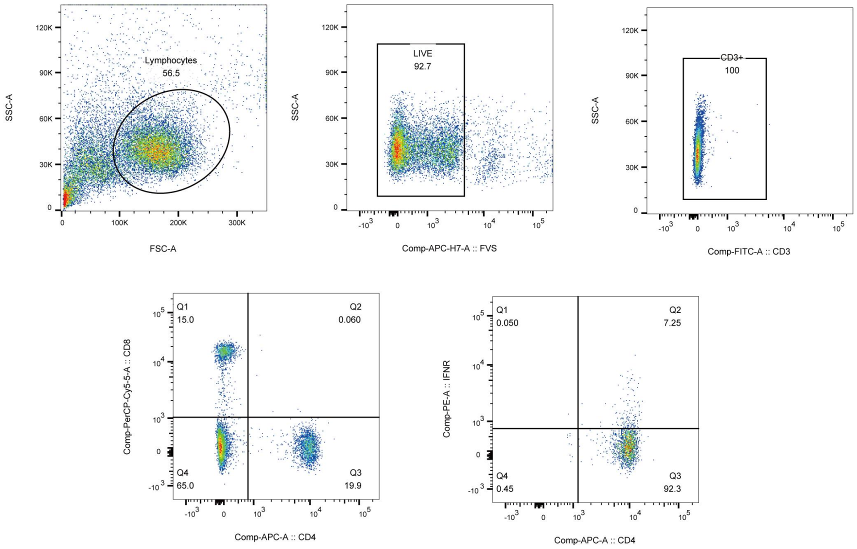

Supplement: Supplementary file 1 [file Data_Sheet_1.PDF]
